# p53 SUMOylation Mediates AOPP-Induced Endothelial Senescence and Apoptosis Evasion

**DOI:** 10.3389/fcvm.2021.795747

**Published:** 2022-02-03

**Authors:** Yanjia Chen, Zhuanhua Liu, Hongyu Chen, Xingfu Huang, Xiaoxia Huang, Yang Lei, Qing Liang, Jiayi Wei, Qin Zhang, Xiaohua Guo, Qiaobing Huang

**Affiliations:** ^1^Department of Pathophysiology, Guangdong Provincial Key Laboratory of Shock and Microcirculation, School of Basic Medical Sciences, Southern Medical University, Guangzhou, China; ^2^Department of Anesthesiology, Nanfang Hospital, Southern Medical University, Guangzhou, China; ^3^Department of Cardiology, Nanfang Hospital, Southern Medical University, Guangzhou, China

**Keywords:** p53 SUMOylation, endothelial senescence, evasion of apoptosis, autophagy, vascular disease

## Abstract

The aging of endothelial cells plays a critical role in the development of age-related vascular disease. We established a model of endothelial premature senescence by application of Advanced oxidation protein products (AOPPs) modified bovine serum albumin (AOPP-BSA) in human umbilical vein endothelial cells (HUVECs). This cellular senescence was accompanied with endothelial barrier dysfunction and angiogenesis impairment. It was further revealed that these senescent HUVECs underwent apoptosis evasion and the receptor for advanced glycation endproducts (RAGE) played a role in these processes. The AOPP-induced senescence was regulated by the state of autophagy in HUVECs. We further proved that AOPP-BSA attenuated the autophagy of HUVECs, led to p53 SUMOylation at K386, resulting in endothelial senescence. We also established the animal model of vascular senescence by using ApoE^−/−^ mice fed with high-fat diet plus daily injection of AOPP-BSA to verify the role of p53 SUMOylation in vascular senescence. Combined with intraperitoneal injection of rapamycin, the effect of autophagy on AOPP-induced p53 SUMOylation was also confirmed *in vivo*. Our data indicates that p53 SUMOylation at K386 plays an important role in AOPP-induced endothelial senescence and apoptosis evasion, suggesting that p53 K386 SUMOylation may serve as a potential therapeutic target in protecting against vascular senescence.

## Introduction

Vascular senescence contributes to the initiation and progression of vascular disease, leading to a variety of related diseases, such as hypertension, coronary heart disease, stroke, and peripheral arterial disease, which cause extremely high morbidity and mortality worldwide. Cellular senescence, especially the aging of endothelial cells, is one of the major reasons of vascular senescence (1). Many studies have demonstrated that endothelial senescence occurs in the development of atherosclerosis and hypertension (2–4). Endothelial dysfunction caused by endothelial senescence is the pathophysiological basis for the development of various vascular diseases (5).

Advanced oxidation protein products (AOPPs) are the products of cellular oxidative stress, accumulated gradually with age and increased in the serum of patients with vascular disease (6, 7). Our preliminary study found that AOPPs induced premature senescence of endothelial cells. Therefore, we first established the endothelial premature senescence model by AOPP treatment to explore the potential molecular mechanism of cellular senescence.

Whether senescent cells undergo apoptosis or escape from apoptosis and what determines the fate of senescent cells, these issues have aroused academic controversy. At present, most studies on apoptosis evasion focus on tumors, while studies on endothelial evasion of apoptosis are scarce (8–10). Due to the low replacement rate of endothelial cells in the human body, it is challenging to regenerate from injury. We assumed that the senescent endothelial cells are likely to escape from apoptosis rather than undergoing apoptosis, surviving in a senescent state for a long time, and accumulate in the body, thus causing vascular senescence and dysfunction. Therefore, it is of great significance to clarify whether senescent endothelial cells escape from apoptosis and elucidate its mechanism for the prevention and treatment of aging-related diseases.

Previous studies on cellular senescence were mostly related to telomerase and Sirt-mediated acetylation (3, 11–13). However, our preliminary study showed that p53 SUMOylation increased in senescent endothelial cells. Besides, many other studies suggest that the level of p53 and p53 post-translational modification play a key role in the fate determination of senescent cells (14–17). As a reversible post-translational modification, the role of p53 SUMOylation in endothelial senescence and apoptosis evasion is still unclear. Therefore, our study aimed to explore the role of p53 SUMOylation in mediating AOPP-induced endothelial senescence and apoptosis evasion, and thus provide a novel target for treatment of vascular senescence associated disease.

## Materials and Methods

### Preparation of AOPP-BSA

AOPP-BSA was prepared as previously reported (18). Briefly, sterile BSA was incubated with HOCl at a 1:140 molar ratio at room temperature for 30 min by a light-free reaction, while the control albumin was incubated without HOCl. After the incubation period, both solutions were dialyzed overnight against sterile PBS to remove free HOCl. Contaminated endotoxin was removed by using Detoxi-Gel column (Thermo Scientific). The endotoxin in the samples was measured with the amebocyte lysate assay kit (Sigma-Aldrich) and was <0.25 EU/ml. The content of AOPP was determined by chloramine-T equivalents (μmol/L) as reported. In brief, 200 μl of the sample with 10-fold dilution was added to a 96-well plate and mixed with 20 μl of acetic acid. In standard wells, 200 μl of different concentration of chloramine-T solution (0–100 μmol/L) was mixed with 10 μl of 1.16 mol/L potassium iodide followed by the addition of 20 μl of acetic acid. The absorbance of the reaction mixture was immediately read by a microplate reader at 340 nm, showing that AOPP-BSA contained an AOPP content of 70.2 ± 1.71 nmol/mg protein, while BSA contained an AOPP content of 0.36 ± 0.02 nmol/mg protein.

### Measurement of Transendothelial Electrical Resistance

Transendothelial electrical resistance (TER) of HUVEC monolayer was measured with EVOM^2^ (World Precision Instruments). Briefly, 200 μl of cells at 1 × 10^5^/well were seeded onto rat-tail collagen (Corning)-coated upper chamber of a trans-well filter (Corning) with 0.4 μm pore size. When the endothelial cells grew to form confluent monolayers, TER of endothelial monolayers were measured before and after AOPP-BSA treatment respectively. The mean value of TER was recorded in the common unit (Ω cm^2^) after subtraction of the value of a blank trans-well insert. The relative changes of TER to baseline value were calculated by the formula: TER = TER of experimental wells/baseline TER of experimental wells-1. All experiments were done in triplicate and repeated at least three times.

### Measurement of Dextran Transendothelial Flux

HUVECs (ScienCell) at 1 × 10^5^/well were plated at the trans-well filter as mentioned in TER assay. When HUVECs grew to 100% confluence, the tracer FITC-labeled dextran (Sigma-Aldrich, average molecular weight 40 kDa) was added to the top chamber and incubated for 45 min at 37°C after AOPP-BSA treatment. Then the concentration of FITC-dextran in upper and bottom chamber was determined with a multiscan spectrum (Spectra Max M5, Molecular Devices). Finally, we calculated Pa to evaluate the endothelial monolayer permeability: Pa = [A]/t × 1/A × V/[L], where [A] represents the dextran concentration in bottom chamber, t refers to time (s), A indicates the area of the filer membrane (cm^2^), V is the volume of the bottom chamber, [L] is the dextran concentration in upper chamber. Then we obtained the ratio of experimental wells Pa value to the control wells Pa value, that is, Pa% = (Pa value of the experimental group/Pa value of the control group) × 100. Pa% is proportional to the permeability of endothelial cells.

### Cell Counting Kit-8 Assay

Cell counting kit-8 assay (Invigentech) was used for cell proliferation evaluation. According to the manufacturer's instructions, 1 × 10^4^/well of HUVECs were seeded in a 96-well plate and grew to 80% confluence, then 10 μl CCK-8 solution was added into each well after AOPP-BSA treatment. HUVECs were incubated with CCK-8 solution for 2 h at 37°C and the absorbance of each well was measured with a microplate reader at 450 nm. Each test was repeated at least three times.

### Trans-well Migration Assay

HUVECs were seeded into the upper trans-well chamber with 8 μm pore size (Corning). In the bottom chamber, 800 μl of AOPP-BSA with serum-free medium was added and incubated for different time at 37°C in a humidified incubator with 5%CO_2_. After AOPP-BSA treatment, the lower side of the trans-well chamber was fixed with 4% paraformaldehyde and stained with 2% crystal violet for 10 min. The cells on the upper side of the trans-well chamber were wiped off with a cotton swab. The number of migration cells was counted under a microscope in three random visual fields, and all experiments were repeated in triplicate.

### Matrigel Tube Formation Assay

The 96-well plate was precoated with 50 μl of Matrigel Basement Membrane Matrix (Corning) and was placed in a incubator with 37°C for 30 min. HUVECs were seeded at a density of 2 × 10^4^ onto each well and incubated at 37°C in a incubator with 5% CO_2_ for 24 h. At different time point with AOPP-BSA treatment, the average number of capillary-like branching points and tube length was counted in three random microscopic fields. All experiments were repeated at least three times.

### RAGE SiRNA Transfection and TP53 Adenovirus Infection

According to the protocol provided by GenePharma (Shanghai, China), HUVECs were transfected with siRNA (20 μM) in serum-free medium using siRNA-Mate when HUVECs cultured to 30–50% confluence. Forty-eight to seventy-two hours after transfection, cells were treated with or without AOPP-BSA and then lysed and subjected to western blot.

HUVECs were plated in culture plates for overnight and grew to 40–60% confluence for adenovirus infection according to the manufacturer's instruction. Then HUVECs were incubated with adenovirus for 8–12 h at 37°C with 5%CO_2_. Afterwards, the solution containing the adenovirus was removed and HUVECs were cultured with complete medium for about 48 h. Cells were observed everyday with fluorescence microscope after infection and proteins extracted from cells were detected by western blot after AOPP-BSA treatment.

### Senescence-Associated Beta-Galactosidase Assay

Senescence-associated beta-galactosidase (SA-β-gal) assay was used to identify the senescent cells. HUVECs were plated in 35 mm dishes and cultured to 100% confluence. After AOPP-BSA treatment, HUVECs were fixed at room temperature for 15 min with 1 ml of specific fixation solution in SA-β-gal staining kit (Beyotime), then washed twice with PBS, and incubated with staining solution at 37°C in a CO_2_-free incubator for 12–16 h. Finally, the number of SA-β-gal positive cells were counted with microscope in three random visual fields.

### Annexin V-FITC/PI Assays

The Annexin V-FITC/PI assays with flow cytometry were used for apoptosis detection. In brief, HUVECs were collected 12 h after AOPP-BSA treatment and resuspended with 400 μl Annexin V-binding buffer (BestBio), following by incubation with 5 μl Annexin V-FITC for 15 min and 10 μl propidium iodide (PI) for 5 min at room temperature in the dark. About 1 5 × 10^5^ cells per tube were collected for detection, and apoptosis in HUVECs was quantitated by flow cytometry (BD FACSVerse^TM^, BD Biosciences) and analyzed by Flowjo software.

### Animal Models

The ApoE^−/−^ mice (B6/JGpt-*Apoe*^*em*1*Cd*82^/Gpt) of about 18–22 g at weight were supplied by GemPharmatech Co., Ltd (Shanghai, China). Mice were maintained at an environmental temperature of 22 ± 2°C with 12:12-h day/night cycles and fed with high-fat diet (22% fat + 0.15% cholesterol) along with free access to drinking water *ad libitum*. Only male mice were used in AOPP-induced model. The ApoE^−/−^ mice aged 4–6 weeks were randomly divided into four groups, and injected intraperitoneally with saline (0.9% NaCl 200 μl), BSA (50 mg/kg/d), AOPP-BSA (50 mg/kg/d), or AOPP-BSA (50 mg/kg/d) + Rapamycin (MedChemExpress, 1 mg/kg/d) for 100 days and 200 days respectively. No mice were excluded from the experiments. At then end of the experiments, mice were anesthetized with 10% chloral hydrate 8 μl/g body weight using intraperitoneal injection. After the opening of abdominal cavity and thoracic cavity of mice along midline, vessels were perfused with saline through left ventricle. Under a stereomicroscope, all adventitial fat of the aorta was removed before the entire aorta was isolated. The aortas were used for further relevant parameter measurement.

### Western Blotting

Proteins were extracted from HUVECs using the RIPA Lysis buffer (Beyotime) supplemented with Protease/Phosphatase Inhibitors Cocktail (TargetMol). For the preparation of aortic tissue homogenate, 300 μl RIPA Lysis buffer with Protease Inhibitors Cocktail and three grinding beads were added to every 10 mg of aortic tissue. The homogenate was prepared by grinder at −30°C running temperature and 75 Hz frequency. Then samples were centrifuged at 12,000 rpm for 15 min at 4°C. The sediments were discarded, and the grinding beads were recovered, the supernatant was used as sample protein.

HUVECs or aortic tissue protein samples were separated in SDS-PAGE gel, and then transferred to polyvinylidene difluoride membranes (Merck Millipore). After blocked with 5% Bovine Serum Albumin, the membranes were incubated respectively with primary antibodies for anti-p16 (ABclonal, catalog A0262), anti-p21 (ABclonal, catalog A2691), anti-LC3A/B (Cell Signaling Technology, catalog 12741), anti-Beclin 1 (Proteintech, catalog 11306-1-AP), anti-p62 (ABclonal, catalog A7758), anti-RAGE (Abcam, catalog Ab216329), anti-p53 (Immunoway, catalog YT3528), anti-Acetyl-p53 (Immunoway, catalog YK0017), or anti-SUMO1 (Immunoway, catalog YT4470) at 1:1,000 at 4°C overnight, washed three times with each time for 10 minutes, and then incubated with secondary antibody at 1:5,000 for 1 h at room temperature. Protein bands were finally visualized with chemiluminescence and densitometric analysis was operated by an imaging station.

### Immunoprecipitation

HUVEC or aortic tissue proteins were prepared using the RIPA Lysis buffer for immunoprecipitation containing Protease/Phosphatase Inhibitors Cocktail. According to the immunoprecipitation protocol, 0.5–1.0 μg of anti-p53 antibody (Proteintech, catalog 60283-2-Ig) was added to 0.5–1.0 ml of lysate sample prepared from 10^6^ to 10^7^ cells typically and the anti-p53 antibody-antigen reactions were completed at 4°C overnight on a rocking platform. Then 40 μl of Protein A+G Agarose (Beyotime) was added to the antibody-antigen lysate mentioned above, and gently incubated on a rocking platform for 1 h at 4°C. The Protein A+G Agarose was collected by centrifugation at 1,000 g for 5 min at 4°C, and the immune complexes in PBS were washed to remove nonspecific binding. The immune complexes were washed five times, and the final wash was removed as completely as possible. In the end, the samples were added the SDS loading buffer and heated at 100°C for 5 min to release both noncovalently bound anti-p53 antibodies and antibody fragments along with p53. Then the levels of p53 and p53 SUMOylation were detected by western blot immediately.

### Immunofluorescent Test

HUVECs were plated in petri dish and cultured till fully confluent. After AOPP-BSA stimulation, HUVECs were washed three times with PBS and fixed with 4% paraformaldehyde for 10 min and then permeabilized with 0.1% Triton X-100 at room temperature for 5 min. HUVECs were blocked with 5% BSA before incubated with anti-LC3A/B (Cell Signaling Technology, catalog 12741, 1:100) at 4°C overnight. Cells were stained with florescent Alexa Fluor-conjugated secondary antibody (Immunoway, catalog RS3611, 1:200) at room temperature for 1 h after washed with PBS and then incubated with DAPI (Solarbio, catalog C0065) to label nuclear DNA. In the end, HUVECs were washed three times in PBS and photographed with a Zeiss LSM780 laser confocal scanning microscope (Zeiss).

The isolated aortas of ApoE^−/−^ mice was removed immediatedly, fixed with 4% paraformaldehyde and then embedded in paraffin. The aorta tissue was then sectioned with deparaffinization and antigen retrieval were performed before primary antibody incubation. Sections were stained by RAGE (1:400, Abcam, catalog ab3611), LC3A/B (1:200, CST, catalog 12741), SUMO1 (1:300, Immunoway, catalog YT4470), p16 (1:80, abclonal, catalog A0262) or p21 (1:150, abclonal, catalog A2691) with CD31 (1:1500, Servicebio, catalog GB11063-2) overnight at 4°C in a humidified chamber, then incutated with corresponding secondary antibody CY3 or FITC for light avoidance at room temperature. After a 5-min washing with PBS three times, cell nucleus were stained with DAPI (Solarbio, catalog C0065) for 10 min. Fluorescence was finally observed using a Zeiss LSM780 laser confocal scanning microscope (Zeiss).

### Immunohistochemical Staining

The isolated aortas of ApoE^−/−^ mice was removed immediatedly, fixed with 4% paraformaldehyde and then embedded in paraffin. The aorta paraffin block was sectioned at 4 μm and incubated with primary antibody against p16, LC3A/B (Cell Signaling Technology, catalog 12741, 1:100), RAGE (Abcam, catalog Ab216329, 1:100), or SUMO1 (Immunoway, catalog YT4470, 1:200) at 4°C overnight after deparaffinization and antigen retrieval, following by secondary antibody incubation at room temperature for 1 h. After three washes in Tris buffer, DAB substrate working solution was used to visualize the antigen/antibody complex. Finally, aorta images were photographed with Zeiss Imager Z2 microscope (Zeiss) and analyzed by ImageJ for positive area.

### Oil Red O Staining

For Oil Red O staining, all adventitial fat of the aorta from ApoE^−/−^ mice in each group was removed under a stereomicroscope and then the entire aorta was carefully separated and fixed with 4% paraformaldehyde for 24 h. Subsequently, the aortas were stained with Oil Red O for 10 min at room temperature for the lipid accumulation analysis. The plaque areas were analyzed by Image-Pro Plus Software.

### Statistical Analysis

All data were analyzed using SPSS 20.0 statistical software and expressed as mean ± SEM of at least three independent experiments. Student's *t* test, one-way ANOVA and two-way ANOVA were used for statistical analysis. LSD *post hoc* analysis was used to compare data among multiple groups when equal variances assumed while Dunnett's T3 test was adopted when not assumed. *P* < 0.05 was considered statistically significant.

## Results

### AOPP-BSA Induces Endothelial Senescence and Apoptosis Evasion

**Figure 1:**
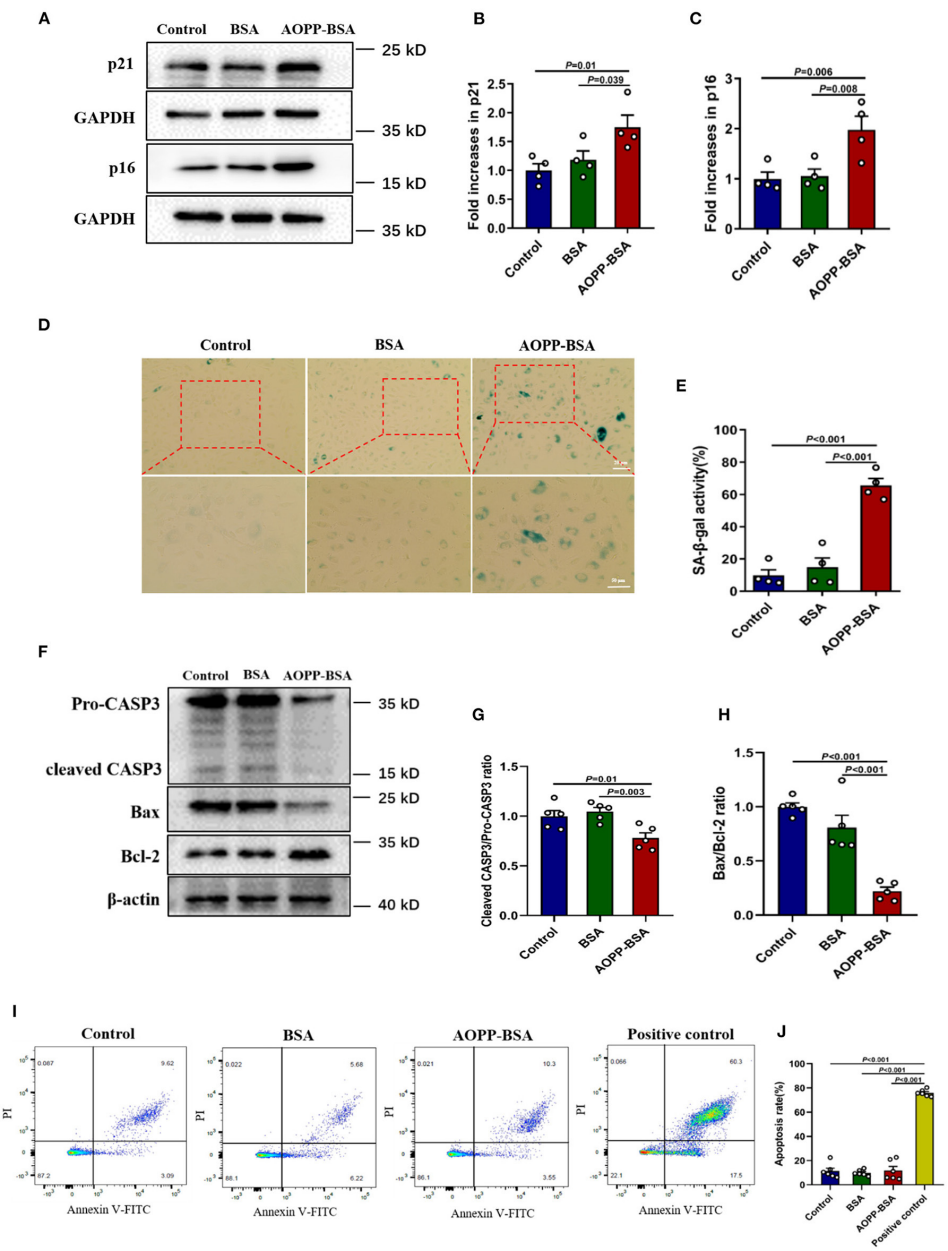
AOPP-BSA mediates endothelial senescence and apoptosis evasion. HUVECs were treated with 200 μg/ml AOPP-BSA for 12 h. Serum-free medium was considered as blank control and BSA was applied as negative control. **(A)** The expression of senescence-associated proteins p21 and p16 were detected by western blot. GAPDH was used as loading control. **(B,C)** Quantification of p21 and p16 protein expression (*n* = 4). **(D)** The senescent cells was identified with SA-β-gal assay. Scale bar for upper panel = 20 μm, scale bar for lower panel = 50 μm. **(E)** The SA-β-gal positive rate was quantitated (*n* = 4). **(F)** The expression of apoptosis-related proteins CASP3, Bax, and Bcl-2 were detected by western blot. β-actin was used as loading control. **(G,H)** The changes of cleaved CASP3/Pro-CASP3 ratio and Bax/Bcl-2 ratio were compared (*n* = 5). **(I)** Apoptosis rate in HUVECs was detected by Annexin V-FITC/PI assays with flow cytometry. **(J)** Apoptosis rate was compared (*n* = 6). HUVECs exposed to the temperature of 55°C for 10 min were used as apoptosis positive control. One-way ANOVA with LSD or Dunnett's T3 *post-hoc* multiple comparisons was used for statistical analysis. All data are shown as mean ± SEM. CASP3, caspase 3.

AOPPs are accumulated gradually with age and it is likely to be an important factor leading to cellular and vascular senescence (19, 20). The effects of AOPP-BSA on endothelial senescence were first evaluated by western blot and SA-β-gal assay in human umbilical vein endothelial cells (HUVECs). The results showed that, compared with control group, AOPP-BSA increased the expression of senescence-related proteins p21 and p16, as well as SA-β-gal activity in HUVECs (Figures 1A–E). Therefore, HUVECs stimulated by AOPP-BSA were used as the premature senescent cell model in our study to explore the potential molecular mechanism of endothelial senescence.

To elucidate whether senescent cells undergo apoptosis or escape from apoptosis, we detected the cellular apoptosis rate by Annexin V-FITC/PI assays and the expression of apoptosis-related protein by western blot and compared the changes of pro- and anti-apoptotic protein ratio. We found that AOPP-BSA up-regulated the expression of Bcl-2, down-regulated the expression of Bax and the ratio of Bax/Bcl-2 was significantly decreased. The activation of apoptotic executive protein caspase 3 was evaluated with a decrease of ratio of cleaved-caspase 3/pro-caspase 3 (Figures 1F–H). The subsequent unchanged apoptosis rate of endothelial cells confirmed that AOPP-BSA mediated endothelial senescence and apoptosis evasion as well (Figures 1I,J).

**Figure 2:**
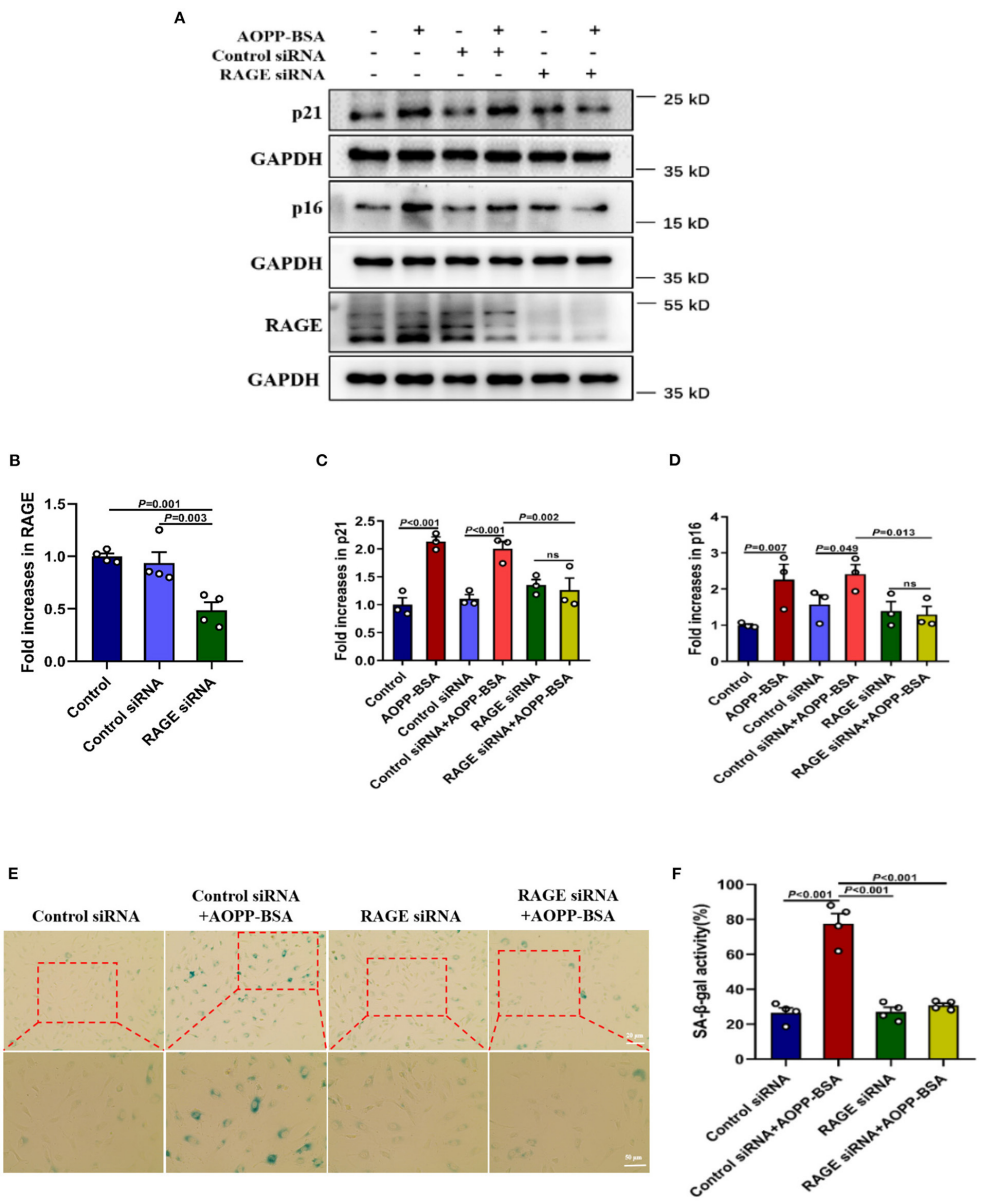
AOPP-BSA promotes endothelial senescence *via* RAGE. **(A)** HUVECs were transfected with RAGE siRNA for 48 h before exposure to AOPP-BSA (200 μg/ml, 12 h). Serum-free medium was used as blank control and control siRNA as negative control. The expression of RAGE, p21 and p16 were detected by western blot. GAPDH was used as loading control. **(B–D)** Quantification of RAGE (*n* = 4), p21 (*n* = 3), and p16 (*n* = 3) protein expression. **(E)** HUVECs were transfected with RAGE siRNA or control siRNA before AOPP-BSA application. The senescent cells was identified with SA-β-gal assay. Scale bar for upper panel = 20 μm, scale bar for lower panel = 50 μm. **(F)** The SA-β-gal positive rate was quantitated (*n* = 4). Data were analyzed by one-way ANOVA with LSD or Dunnett's T3 *post-hoc* multiple comparisons. All data are shown as mean ± SEM. ns, non-significant (*P* > 0.05).

The receptor for advanced glycation endproducts (RAGE) is also regarded as an important receptor for AOPPs (7, 21). To investigate whether RAGE is required for AOPP-induced endothelial senescence, HUVECs were transfected with RAGE siRNA to knock down RAGE protein level before AOPP-BSA application. Our data revealed that RAGE down-regulation attenuated AOPP-BSA-induced up-regulation of p16 and p21 (Figures 2A–D) and prevented AOPP-BSA-induced increase of SA-β-gal activity (Figures 2E,F). These results suggested that AOPP-BSA induced endothelial senescence through RAGE.

### AOPP-BSA Mediates Endothelial Barrier Dysfunction and Impairs Angiogenesis

**Figure 3:**
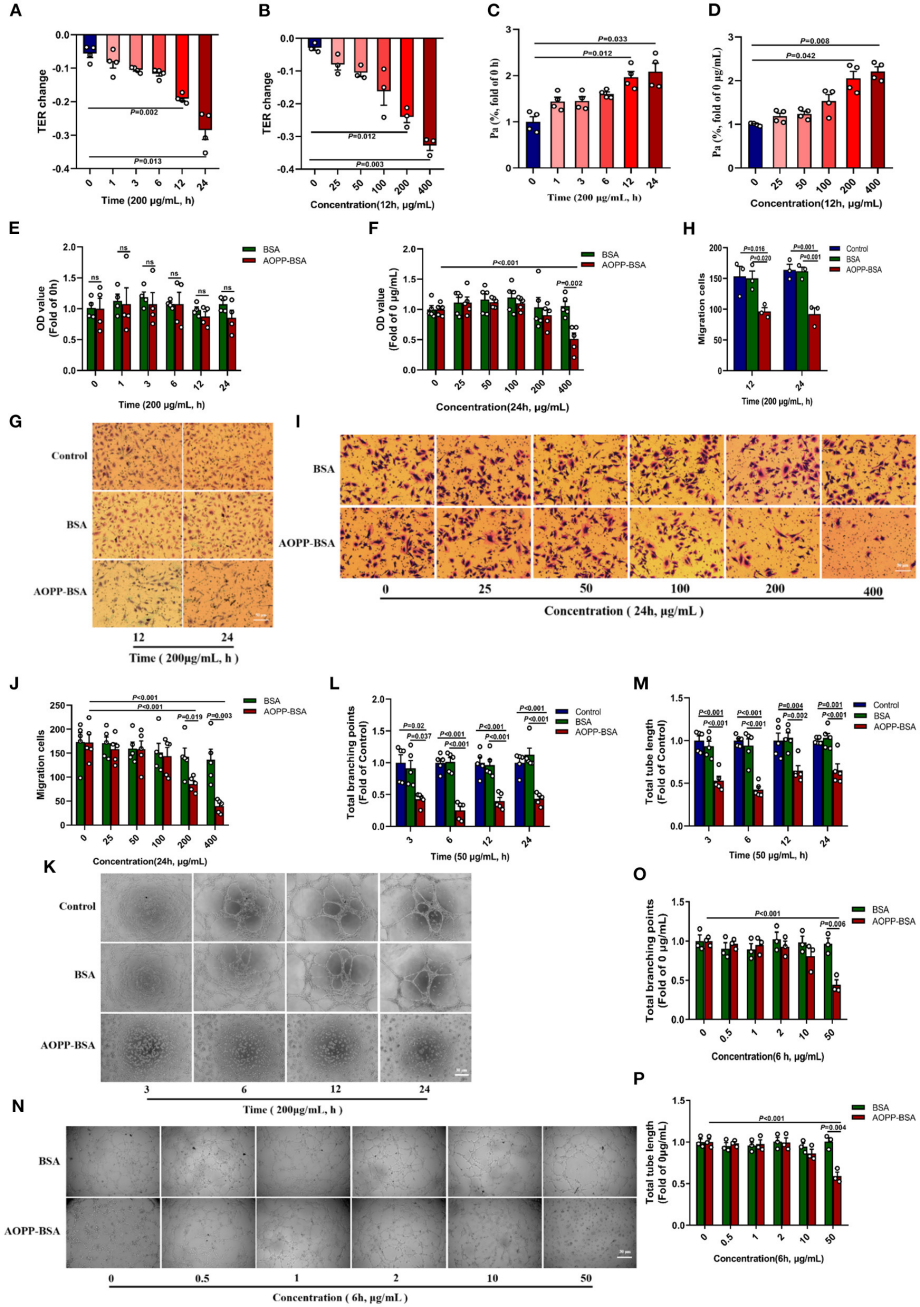
AOPP-BSA mediates endothelial hyperpermeability and impairs angiogenesis. **(A,C)** HUVECs were stimulated with 200 μg/ml AOPP-BSA for 0, 1, 3, 6, 12, 24 h, then transendothelial electrical resistance (TER) and permeability coefficient of FITC-dextran (Pa) were detected (*n* = 4). **(B,D)** HUVECs were treated with AOPP-BSA at 0~400 μg/ml for 12 h, then TER (*n* = 3) and Pa (*n* = 4) were measured. **(E,F)** CCK-8 assay was used to evaluate the proliferation function of HUVECs with AOPP-BSA treatment for different time (*n* = 4) or at different concentrations (*n* = 5). **(G,I)** The migration function of HUVECs treated with AOPP-BSA was detected by trans-well migration assay. Scale bar = 50 μm. **(H,J)** The number of trans-well migration cells was counted (*n* = 3–5). **(K,N)** Matrigel tube formation assay was used for tube formation evaluation in HUVECs incubated with AOPP-BSA. Scale bar = 30 μm. **(L,M)** Tube length and branching points of HUVECs treated with AOPP-BSA for different time were quantitated (*n* = 5). **(O,P)** Tube length and branching points of HUVECs treated with AOPP-BSA at different concentrations were quantitated (*n* = 3). Two-way ANOVA was used for statistical analysis. All data are shown as mean ± SEM. ns, non-significant (*P* > 0.05).

It is reported that the senescence of endothelial cells are accompanied by modulation in cytoskeleton integrity, proliferation, cell migration, and angiogenesis (22, 23). In this study, HUVECs were incubated with different concentrations of AOPP-BSA for different duration. The results showed that TER of HUVECs gradually decreased and Pa gradually increased. The changes of TER and Pa became significant at the concentration of 200 and 400 μg/ml AOPP-BSA and at the duration of 12 and 24 h (Figures 3A–D), which indicated a concentration-dependent and time-dependent increase in endothelial monolayer permeability. In addition, CCK-8 proliferation assay, trans-well migration assay and matrigel tube formation assay were used to evaluate the function of endothelial angiogenesis. With the increase of the concentration and duration of AOPP-BSA stimulation, the proliferation and migration abilities of HUVECs were decreased gradually with significant differences at 400 μg/ml and at 24 h (Figures 3E–J). HUVECs, however, seemed to be more sensitive to AOPP-BSA-induced inhibition of tube formation with significant differences at 50 μg/ml. It was difficult for HUVECs to form a complete tube-like structure at any time point with the treatment of 50 μg/ml AOPP-BSA (Figures 3K–P). Together, these data indicated a time-dependent and concentration-dependent decrease in endothelial proliferation, migration and tube formation induced by AOPP-BSA. The endothelial senescence induced by AOPP-BSA were accompanied with endothelial barrier dysfunction and angiogenesis impairment.

### AOPP-BSA Inhibites Endothelial Autophagy in a Concentration-Dependent and Time-Dependent Manner

**Figure 4:**
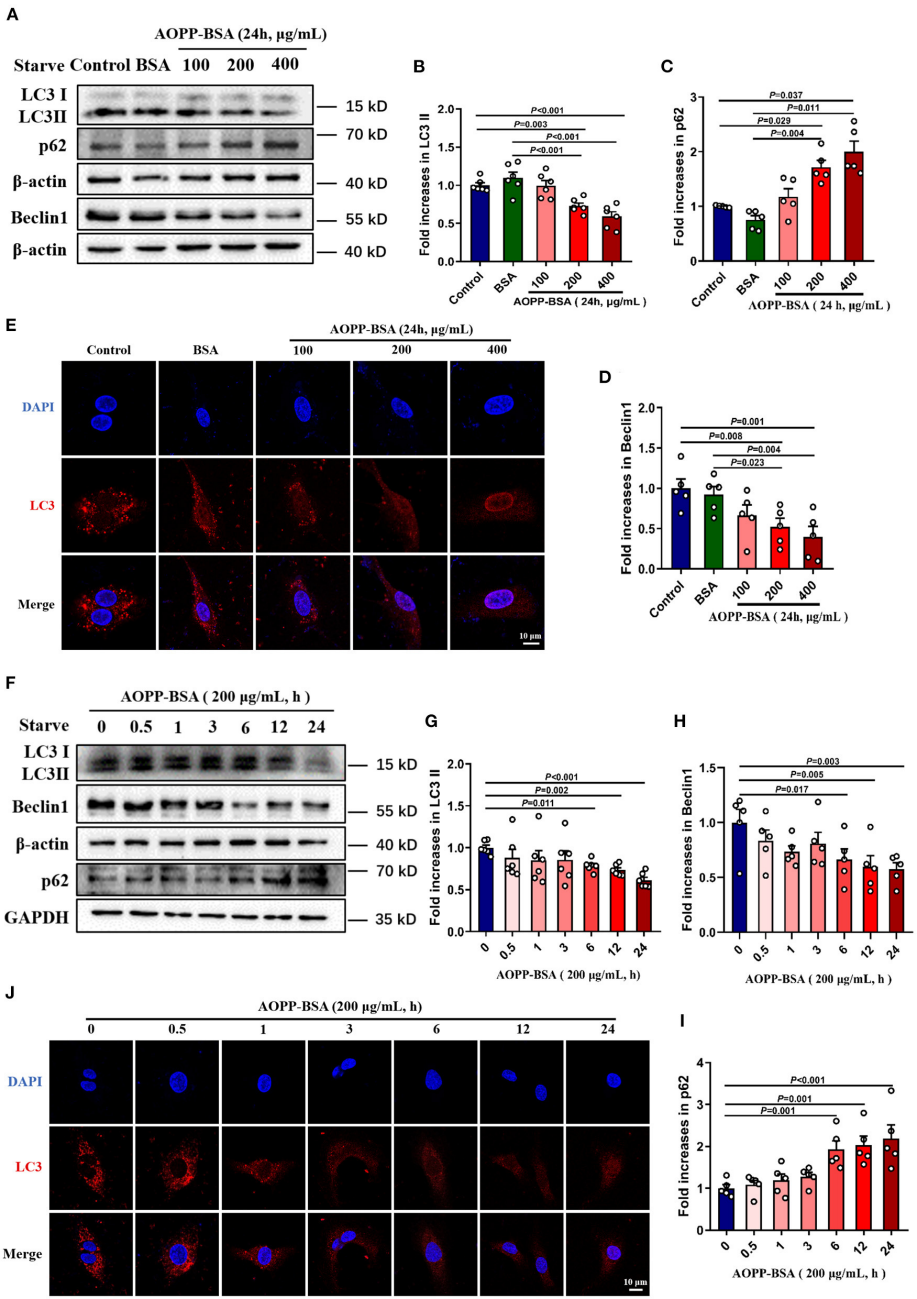
AOPP-BSA attenuates endothelial autophagy. **(A)** HUVECs were treated with AOPP-BSA at 100, 200, and 400 μg/ml for 24 h. Before AOPP-BSA treatment, HUVECs were incubated with Hanks balanced salt solution (HBSS) for 1 h to activate autophagy by starvation. The expression of autophagy-associated proteins LC3II, p62 and Beclin1 were detected by western blot. β-actin was used as loading control. **(B-D)** Quantification of LC3II (*n* = 6), p62 (*n* = 5) and Beclin1 (*n* = 5) protein expression. **(F)** HUVECs were stimulated with 200 μg/ml AOPP-BSA for 0, 0.5, 1, 3, 6, 12 and 24 h. As mentioned above, HUVECs were starved with HBSS for 1 h before AOPP-BSA treatment. The expression of LC3II, p62 and Beclin1 were detected by western blot. β-actin and GAPDH were used as loading control. **(G–I)** Density analysis of LC3II (*n* =6 ), Beclin1 (*n* = 5) and p62 (*n* = 5) protein levels. **(E,J)** The alteration of LC3 (red) level was also observed using immunostaining by a laser confocal microscope in HUVECs and nuclear was indicated with DAPI (blue). Scale bar = 10 μm. One-way ANOVA with LSD or Dunnett's T3 *post-hoc* multiple comparisons was used for statistical analysis. All data are shown as mean ± SEM.

Since autophagy inhibition is thought to be one of the crucial reasons for cellular senescence (24–26), we detected AOPP-BSA-mediated endothelial autophagy by western blot and immunofluorescent test with LC3A/B antibody incubation. The data showed that AOPP-BSA decreased the expression of autophagy-related proteins LC3 II and Beclin 1, and increased the expression of p62 in HUVECs in a concentration-dependent manner with significant differences at 200 and 400 μg/ml (Figures 4A–D), and in a time-dependent manner with significant differences at 6, 12 and 24 h (Figures 4F–I). Immunofluorescence results showed that AOPP-BSA reduced the LC3 punctas in HUVECs in a concentration- and time-dependent manner (Figures 4E,J). The results suggested that AOPP-BSA inhibited endothelial autophagy in a concentration-dependent and time-dependent pattern.

**Figure 5:**
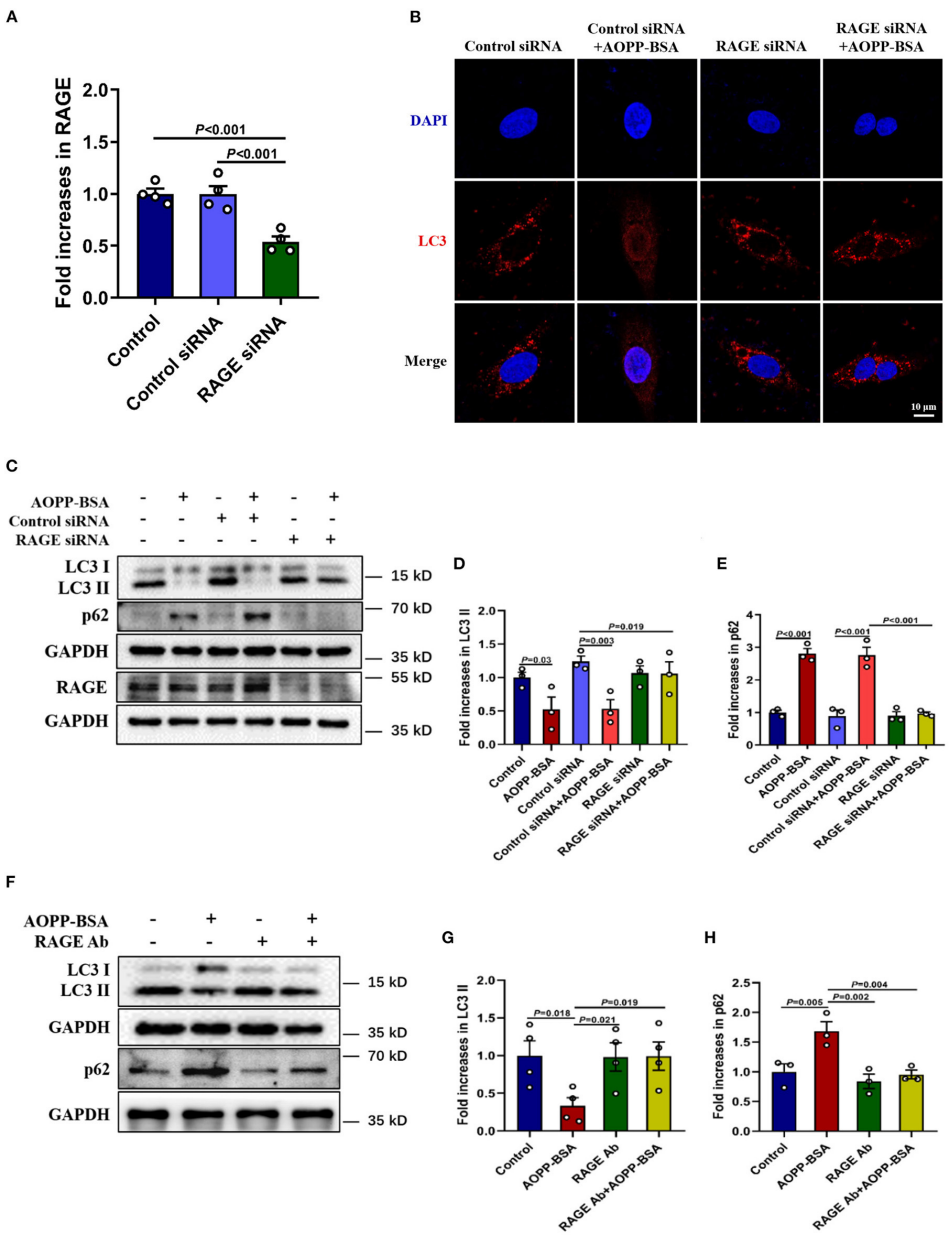
Down-regulation of RAGE alleviates AOPP-BSA-induced autophagy inhibition. **(A)** HUVECs were pretreated with RAGE siRNA and control siRNA for 48 h before AOPP-BSA (200 μg/ml, 12 h) application. The expression of RAGE was detected by western blot and was quantitated (*n* = 4). **(B)** HUVECs were treated as mentioned above, and immunostained with LC3 (red) and DAPI (nuclear, blue). Scale bar = 10 μm. **(C)** HUVECs were treated as mentioned above, then the expression of LC3II and p62 were detected by western blot. GAPDH was used as loading control. **(D,E)** Quantification of LC3II and p62 protein expression (*n* = 3). **(F)** HUVECs were incubated with RAGE neutralizing antibody (RAGE Ab, 10 μg/ml) for 1 h before AOPP-BSA application, then protein levels of LC3II and p62 were detected by western blot. GAPDH was used as loading control. **(G,H)** Quantification of LC3II (*n* = 4) and p62 (*n* = 3) protein expression. One-way ANOVA with LSD or Dunnett's T3 *post-hoc* multiple comparisons was used for statistical analysis. All data are shown as mean ± SEM. RAGE Ab, RAGE neutralizing antibody.

As mentioned above, AOPP-BSA-induced endothelial senescence requires RAGE. To investigate whether RAGE is also required for AOPP-induced inhibition of autophagy, HUVECs were transfected with RAGE siRNA to reduce the expression of RAGE (Figure 5A) or incubated with RAGE neutralizing antibody (RAGE Ab, R&D, catalog MAB11451) to inhibit the function of RAGE before AOPP-BSA stimulation. Our data showed that the reduction of LC3 punctas in AOPP-BSA-treated HUVECs was abolished by RAGE siRNA (Figure 5B). The up-regulation of p16 and p21 induced by AOPP-BSA were also attenuated in RAGE-knockdown or RAGE-inhibition HUVECs (Figures 5C–H).

### AOPP-BSA Mediates Endothelial Senescence Through Autophagy Inhibition

**Figure 6:**
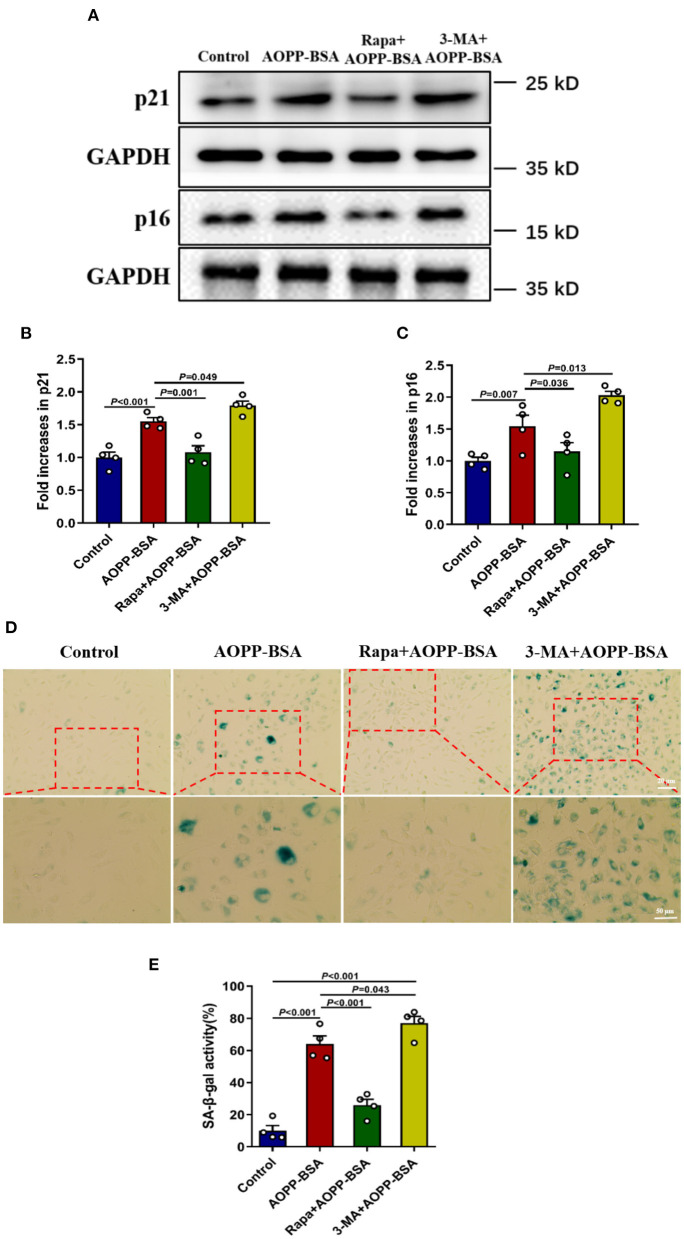
Autophagy inhibition leads to AOPP-BSA-induced endothelial senescence. **(A)** Effects of autophagy activation with Rapa and inhibition with 3-MA on AOPP-BSA-induced expression of senescence-associated protein p21 and p16 were measured by western blot. HUVECs were pretreated with Rapa (200 nM) or 3-MA (5 mM) for 2 h before AOPP-BSA stimulation. GAPDH was used as loading control. **(B,C)** Quantification of p21 and p16 protein expression (*n* = 4). **(D)** HUVECs were pretreated as mentioned above. Effects of Rapa and 3-MA on HUVEC senescence were also detected by SA-β-gal assay. Scale bar for upper panel = 20 μm, scale bar for lower panel = 50 μm. **(E)** The SA-β-gal positive rate was quantitated (*n* = 4). All data analyzed by one-way ANOVA with LSD or Dunnett's T3 *post-hoc* multiple comparisons are shown as mean ± SEM. Rapa, rapamycin; 3-MA, 3-methyladenine.

To explore the role of autophagy in AOPP-BSA-induced endothelial senescence, cells were pretreated with autophagy agonist Rapamycin (Rapa) or autophagy inhibitor 3-methyladenine (3-MA) before AOPP-BSA application. The results revealed that autophagy activation with Rapa attenuated AOPP-BSA-induced p21 and p16 up-regulation in HUVECs and also significantly blocked AOPP-BSA-induced SA-β-gal activation. On the contrary, inhibition of autophagy with 3-MA enhanced AOPP-BSA-induced p21 and p16 up-regulation in HUVECs and also significantly exacerbated AOPP-BSA-induced SA-β-gal activation (Figure 6). These results indicated that AOPP-BSA induced endothelial senescence *via* inhibition of autophagy.

### The Inhibition of Autophagy Enhances AOPP-BSA-Induced p53 SUMOylation

**Figure 7:**
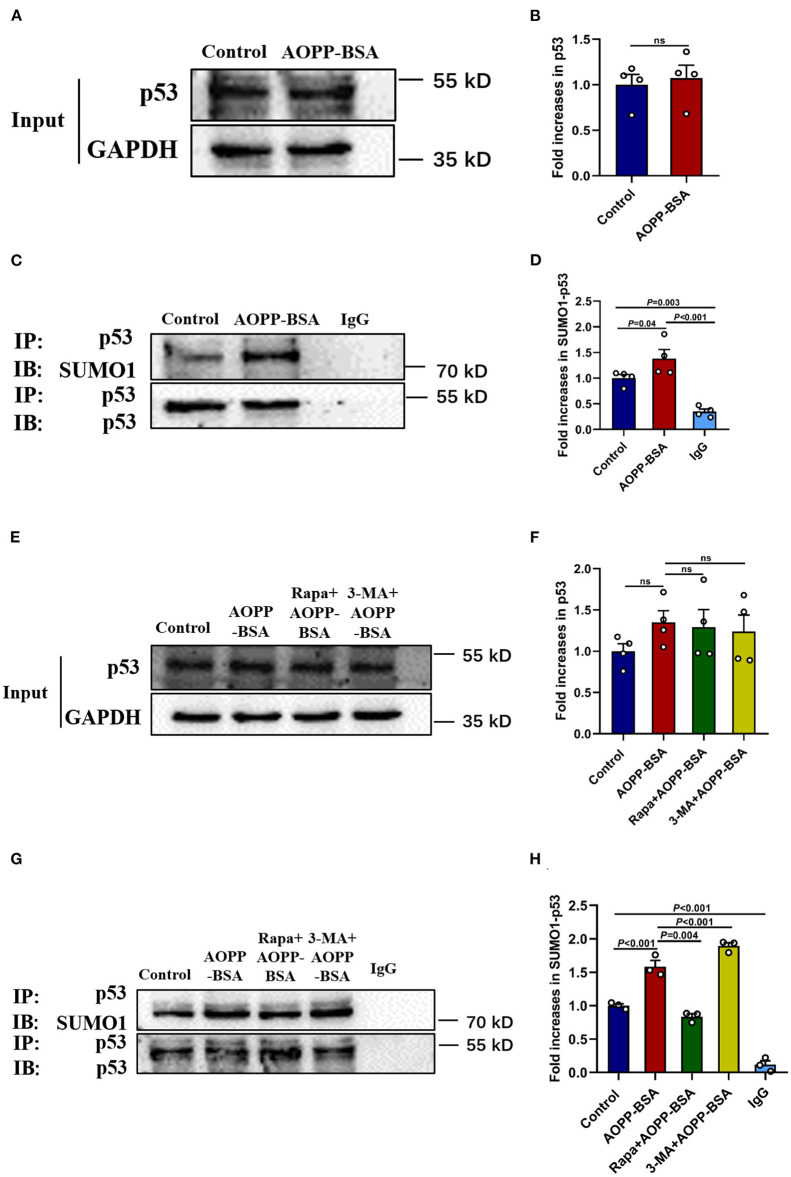
Autophagy inhibition enhances p53 SUMOylation induced by AOPP-BSA. **(A,C)** HUVECs were incubated with 200 μg/ml AOPP-BSA for 12 h. Effects of AOPP-BSA on p53 and p53 SUMOylation were detected by western blot and immunoprecipitation. **(B)** Quantification of p53 protein expression (*n* = 4). **(D)** The ratio of SUMOylated p53 and total p53 was calculated (*n* = 4). **(E,G)** HUVECs were pretreated with Rapa (200 nM) or 3-MA (5 mM) for 2 h before AOPP-BSA application, and the levels of p53 and p53 SUMOylation were detected by western blot and immunoprecipitation. **(F)** Quantification of p53 protein expression (*n* = 4). **(H)** The ratio of SUMOylated p53 and total p53 was calculated (*n* = 3). Two-tailed unpaired Student's *t*-test for **(B)**. One-way ANOVA with LSD or Dunnett's T3 *post-hoc* multiple comparisons for **(D,F,H)**. Data are shown as mean ± SEM. ns, non-significant (*P* > 0.05). SUMO1, small ubiquitin related modifier 1; SUMO1-p53, SUMOylated p53; IP, immunoprecipitation; IB, immunoblotting.

p53 is reported to play a key role in the fate determination of senescent cells. As one of the reversible post-translational modification, SUMOylation has been implied to be involved in the process of cellular senescence. However, the role of p53 SUMOylation in cellular senescence, expecially in endothelial senescence remains unclear. To clarify the role of p53 SUMOylation in AOPP-BSA-induced endothelial premature senescence, HUVECs were treated with AOPP-BSA (200 μg/ml) for 12 h, then the level of p53 and p53 SUMOylation were detected by western blot and immunoprecipitation. We found that p53 SUMOylation was increased significantly in AOPP-BSA-treated HUVECs, but the expression of p53 was unchanged (Figures 7A–D). We further investigated the relationship between autophagy and p53 SUMOylation by Rapa or 3-MA pretreatment before AOPP-BSA application in HUVECs. We observed that the increase of AOPP-BSA-mediated p53 SUMOylation was inhibited by Rapa but enhanced by 3-MA. However, Rapa and 3-MA did not changed the expression of p53 (Figures 7E–H). These findings revealed that AOPP-BSA-induced p53 SUMOylation might be mediated by autophagy inhibition.

### AOPP-BSA Mediates Endothelial Senescence Through p53 K386 SUMOylation

**Figure 8:**
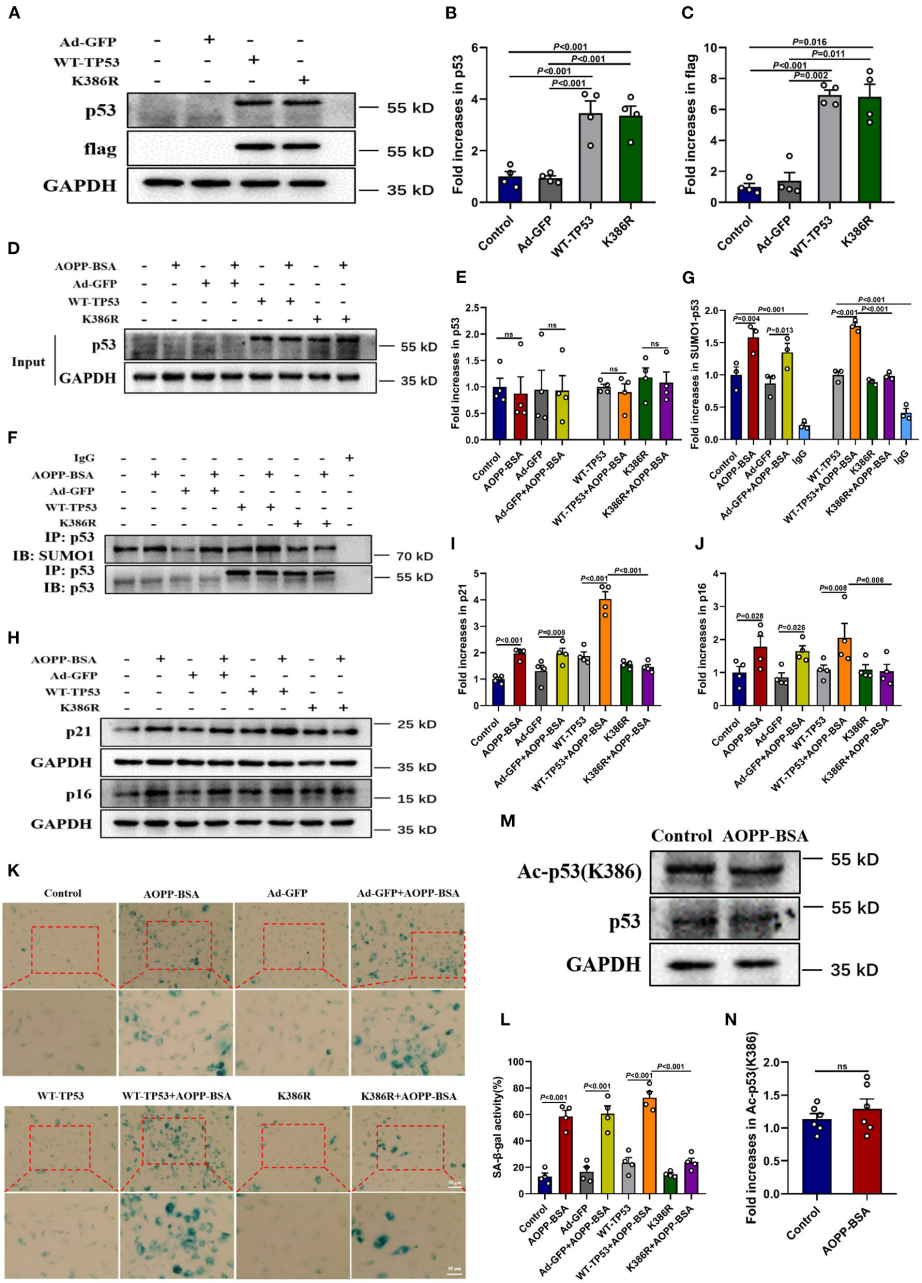
p53 SUMOylation at K386 mediates AOPP-BSA-induced endothelial senescence. **(A)** HUVECs were infected with adenovirus of K386R, WT-TP53, or Ad-GFP for 48 h. The effects of K386R, WT-TP53, and Ad-GFP on the levels of p53 and flag were detected by western blot. GAPDH was used as loading control. **(B,C)** Quantification of p53 and flag protein expression (*n* = 4). **(D,E)** At 48 h post-infection, HUVECs were incubated with or without 200 μg/ml AOPP-BSA for 12 h. The protein levels of p53 and p53 SUMOylation were detected by western blot and immunoprecipitation. **(F)** Quantification of p53 protein expression (*n* = 4). **(G)** The SUMO1-p53/p53 ratio was calculated (*n* = 3). **(H)** HUVECs were infected with K386R, WT-TP53, and Ad-GFP with or without AOPP-BSA stimulation. The expression of senescence-associated proteins p21 and p16 were detected by western blot. **(I,J)** Quantification of p21 and p16 protein expression (*n* = 4). **(K)** HUVECs were treated as mentioned above, and the SA-β-gal assay was used to identify the senescent cells. Scale bar for upper panel = 20 μm, scale bar for lower panel = 50 μm. **(L)** The SA-β-gal positive rate was quantitated (*n* = 4). **(M)** HUVECs were incubated with or without 200 μg/ml AOPP-BSA for 12 h, then the expression of p53 and acetylation of p53 were detected by western blot. **(N)** The ratio of acetylated p53 and total p53 was calculated (*n* =6 ). Two-tailed unpaired Student's *t*-test for **(N)**. One-way ANOVA with LSD or Dunnett's T3 *post-hoc* multiple comparisons for **(B,C,E,G,I,J,L)**. Data are shown as mean ± SEM. ns, non-significant (*P* > 0.05). Ad-GFP, control adenovirus containing GFP; WT-TP53, wide-type TP53 adenovirus; K386R, TP53 K386-deficient adenovirus; Ac-p53 (K386), acetylated p53 at K386.

To further explore the effect of p53 SUMOylation and its SUMOylating site in AOPP-BSA-induced endothelial senescence (27, 28), HUVECs were infected with TP53 K386-deficient adenovirus (K386R), wide type TP53 adenovirus (WT-TP53) or control adenovirus (Ad-GFP) before exposed to AOPP-BSA. The results showed that AOPP-BSA-induced p53 SUMOylation was decreased by K386R, while unchanged by WT-TP53 (Figures 8A–G). We also confirmed that p53 SUMOylated at K386 with no acetylation (Figures 8M,N). Moreover, the up-regulation of p21 and p16 induced by AOPP-BSA was blocked by K386R (Figures 8H–J), and AOPP-BSA-induced SA-β-gal activity increase was also reduced by K386R in HUVECs (Figures 8K,L). Therefore, we can draw the conclusion that AOPP-BSA promoted endothelial senescence by activating p53 SUMOylation at K386.

### AOPP-BSA Mediates Vascular Senescence Through Autophagy Inhibition

**Figure 9:**
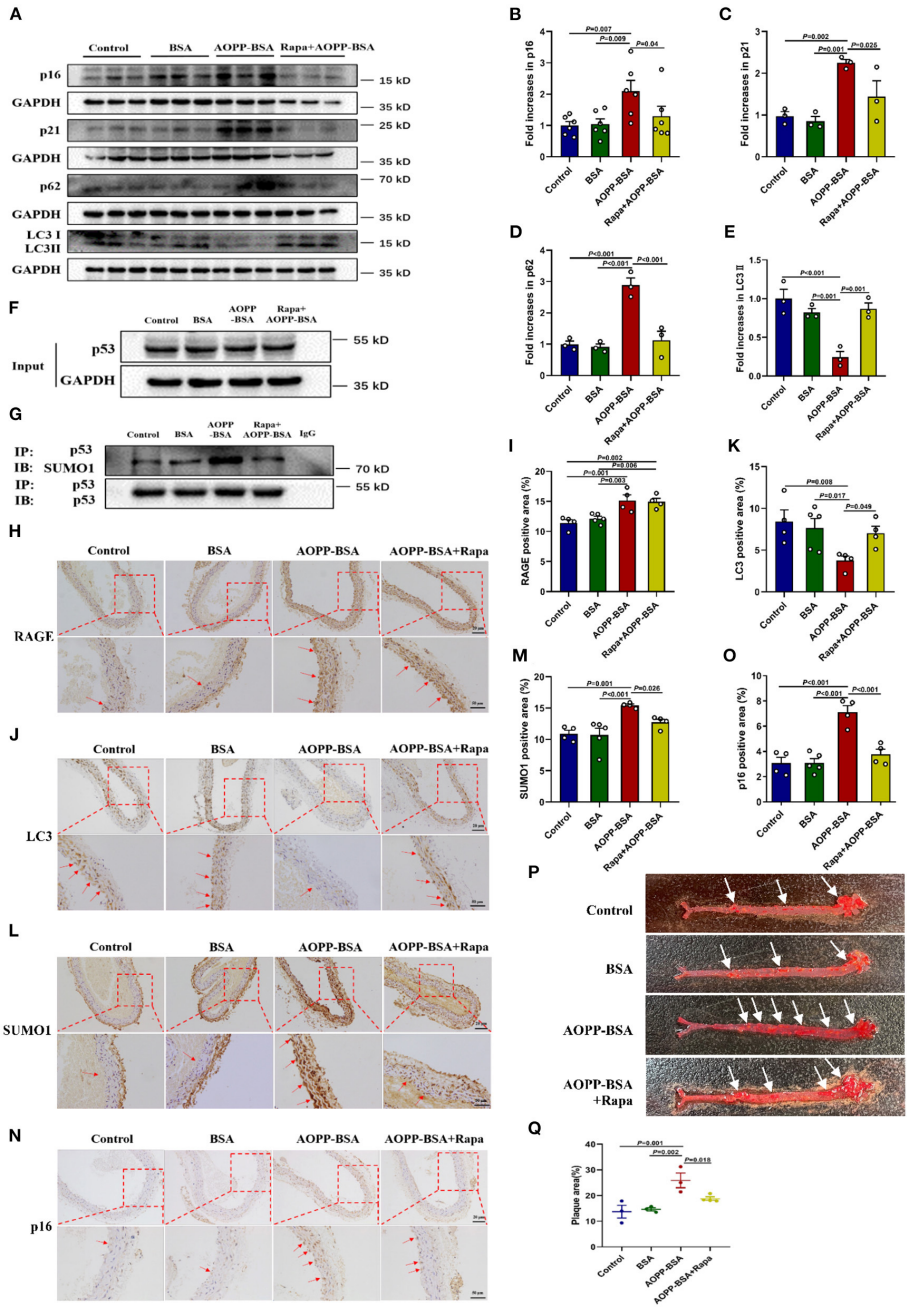
AOPP-BSA promotes vascular senescence through vascular autophagy inhibition. ApoE^−/−^ mice fed with high-fat diet were used to establish the model of vascular_senescence. Saline, BSA (50 mg/kg), AOPP-BSA (50 mg/kg), or AOPP-BSA (50 mg/kg) + Rapa (1 mg/kg) was injected intraperitoneally in 200 μl for consecutive 100 days or 200 days. **(A)** The expression of p16, p21, p62, and LC3II in aortic tissue were detected by western blot. GAPDH was used as loading control. **(B–E)** Quantification of p16 (*n* = 6), p21 (*n* = 3), p62 (*n* = 3), and LC3II (*n* = 3) protein expression. **(F,G)** Immunoprecipitation was used for p53 purification, and the levels of p53 and p53 SUMOylation in aortic tissue were detected by western blot. **(H,J,L,N)** Aortas isolated from mice in different groups were fixed and immunostained with RAGE, LC3, SUMO1 and p16. Images were photographed with Zeiss Imager Z2 microscope (Zeiss, Germany) and analyzed by Image J software. Representative images were showed and red arrows indicated the aortic endothelium. Scale bar for upper panel = 20 μm, scale bar for lower panel = 50 μm. **(I,K,M,O)** Quantification of RAGE, LC3, SUMO1 and p16 positive area in aortic tissue (*n* = 4 in Control group, *n* = 5 in BSA group, *n* = 4 in AOPP-BSA group, *n* = 4 in AOPP-BSA+Rapa group). **(P)** The aortic plaque area of high-fat-fed ApoE^−/−^ mice was revealed by Oil Red O staining and analyzed by Image-Pro Plus Software. Representative images were showed. **(Q)** Quantification of plaque area in aortic tissue (*n* = 3 in Control group, *n* = 3 in BSA group, *n* = 3 in AOPP-BSA group, *n* = 4 in AOPP-BSA+Rapa group). One-way ANOVA with LSD or Dunnett's T3 *post-hoc* multiple comparisons was used for statistical analysis. All data are shown as mean ± SEM.

To verify the conclusions *in vitro*, ApoE^−/−^ mice fed with high-fat diet were used to establish the model of vascular senescence. Saline, BSA, AOPP-BSA, or AOPP-BSA + Rapa was injected intraperitoneally to explore the effect of AOPP-BSA and the role of autophagy in vascular senescence. Results of western blot revealed that AOPP-BSA increased the expression of senescent related proteins p16, p21 in aortic tissue, while autophagy marker protein LC3 II was decreased, accompanied with p62 elevation (Figures 9A–E). Immunoprecipitation test indicated that AOPP-BSA induced vascular p53 SUMOylation (Figures 9F,G). The application of Rapa attenuated all these AOPP-BSA-induced changes in p16, p21, LC3 II, p62 expression and p53 SUMOylation in aortic tissue (Figures 9A–G). Results of immunohistochemical staining unveiled that the application of Rapa had no effect on AOPP-BSA-induced expression of RAGE in aorta (Figures 9H,I). AOPP-BSA exposure lightened the staining of LC3 (Figures 9J,K) and enhanced the staining of p16 (Figures 9L,M), SUMO1 (Figures 9N,O) at the same time, while the application of Rapa attenuated these AOPP-BSA-induced LC3, p16, SUMO1 staining changes in aorta (Figures 9H–O). Furthermore, the colocalizations of RAGE, LC3, SUMO1, p16 and p21 with endothelial cell marker CD31 were observed by immunofluorescence. The results demonstrated that the stainings of RAGE, SUMO1, p16 and p21 were all stronger but that of LC3 was weaker after AOPP treatment and there were colocalization of these proteins with CD31 in the area of vascular intima (Supplementary Figures 1A–E). Results of aorta Oil Red O staining showed that AOPP-BSA increased aortic plaque area in high-fat fed ApoE^−/−^ mice, while Rapa narrowed the plaque area in aortas from mice challenged by AOPP-BSA (Figures 9P,Q). Based on the results above, we concluded that following the autophagy inhibition, AOPP-BSA mediated vascular senescence *via* p53 SUMOylation, thus leading to the acceleration of vascular senescence.

## Discussion

Present study demonstrated that AOPPs could induce endothelial senescence and evasion of apoptosis, accompanying with endothelial dysfunction, including the increase of monolayer permeability, the inhibition of proliferation, migration, and tube formation in HUVECs in time-dependent and concentration-dependent manners. Further experiments suggested that AOPP-induced endothelial senescence might be the consequence of autophagy inhibition and the subsequent p53 K386 SUMOylation in endothelial cells. The experiment in high-fat fed ApoE^−/−^ mouse model confirmed that the senescent endothelial cells accumulated in the vessels could survive through apoptosis evasion, leading to endothelial dysfunction and vascular senescence, eventually resulting in the development of vascular disease.

It has been proved that AOPPs, as products of oxidative stress, could increase the plaque area of aortas in rabbits and thus promote the development of atherosclerosis (20). Clinical data also suggest that the level of AOPPs in the plasma is positively correlated with the development of atherosclerosis-related cardiovascular diseases (29, 30). The results in our study using high fat fed ApoE^−/−^ mice also confirmed that the application of AOPPs enlarged the plaque area in the aorta. However, the specific mechanism by which AOPPs accelerate atherosclerosis is still unclear. A number of studies showed that AOPPs might participate in the development of atherosclerosis through a variety of mechanisms, including but not limited to pro-inflammatory response, pro-oxidative stress, modification of low-density lipoprotein and inhibition of cholesterol reverse transport, etc. For the first time, present study reveals that AOPPs could induce endothelial senescence with serious dysfunction at the same time, suggesting cellular senescence might be an important mechanism for the pathogenic effect of AOPPs.

It has been confirmed that the senescence of endothelial cells undergoes a serious of dysfucntion, such as the disruption of barrier integrity and the inhibitions of proliferation, migration and tube formation of endothelial cells (23, 31). Other studies also proved that vascular dysfunction was closely related to endothelial senescence and thus leading to a wide range of diseases, such as AS, hypertension, and peripheral arterial disease (32). Vascular hyperpermeability and impaired angiogenesis are the outcome of senescence associated endothelial dysfunction, and the senescent endothelium, due to the repair disability, aggravates endothelial injury and promotes the development of vascular diseases (5). In this study, AOPP-induced senescent endothelial cells also suffered from barrier dysfunction. The inhibition of endothelial proliferation suggested that the vascular endothelial repair function was impaired by AOPPs. The attenuation of migration and tube formation of endothelial cells revealed that the function of angiogenesis was also depressed by AOPPs. These results indicate that AOPP exposure would make the blood vessels more fragile and difficult to repair under the stimulation of pathogenic factors. And the depression of angiogenesis would weaken the collateral circulation compensation of ischemic tissue, leading to vascular disease. Interestingly, the AOPP concentration for the inhibition of tube formation was much lower than that required for the inhibition of proliferation and migration, which also suggested that AOPPs induced severe angiogenesis disorders. We assumed that the angiogenesis of endothelial cells was significantly inhibited while the abilities of proliferation and migration were still preserved, which might be related to the imbalanced of pro-angiogenic factors and anti-angiogenic factors in endothelial cells treated with AOPPs.

Whether senescent cells undergo apoptosis or escape from apoptosis is still controversy (14, 33). Our study showed that AOPP-induced endothelial senescence was accompanying with evasion of apoptosis at the same time. At present, most studies on apoptosis evasion focus on cancers. A number of studies have proved that mutated cells could accumulate in the body along with cell proliferation through apoptosis escape, and eventually lead to the development of cancer (34–36). The studies on apoptosis evasion of endothelial cells are scarce. Due to the low replacement rate of endothelial cells in the body, it is challenging to proliferate and repair after injury. Therefore, we assumed that, in response to long-term chronic oxidative stress caused by AOPPs, endothelial cells might escape from apoptosis through initiation of senescence, so as to maintain the basic morphology of blood vessels in structure. However, the decreased viability of senescent endothelial cells would inevitably lead to cellular dysfunction and repair impairment, resulting in the subsequent development of vascular disease.

For the mechanism of AOPP-induced endothelial senescence, present study unveiled that AOPP could mediate the inhibition of autophagy, leading to the SUMOylation of p53 and the subsequent senescence of endothelial cells. Previous studies on cellular senescence were mostly related to telomerase and Sirt-mediated acetylation (12, 37, 38). Studies have shown that the level of p53 and the post-translational modification of p53 play an important role in the fate determination of senescent cells (39). For example, it has been reported that cells from p53^K117R/K117R^mutant mice could not induce apoptosis, but could still undergo cell cycle arrest and senescence through p21 upregulation (40). In this study, by transfecting p53 K386-deficient virus into HUVECs, we demonstrated that AOPPs promoted endothelial senescence by inducing p53 SUMOylation of Lysine at the 386 amino acid residue (K386), while the acetylation of this site K386 was not altered at the same time. Others have reported that SUMOylation was necessary for heterochromatin formation in cellular senescence, and could inhibit the transcription of pro-proliferation genes, which provided support for our research conclusion (41). So we speculate that p53 SUMOylation not only cause the senescence of endothelial cells, but might also mediate senescence of other type of cells. p53 SUMOylation might play an important role in both AOPP-, as well as other biological molecules-induced cellular senescence. In addition, our study showed that inhibition of autophagy could enhance the SUMOylation of p53, while activation of autophagy could decrease the SUMOylation of p53. It has been reported that SUMOylated p53 could transferred from cytoplasm into nucleusm, and then played its role in determining cell fate (42). However, the issue about how autophagy regulates SUMOylation of p53 remains to be elucidated in the future.

One achievement worth mentioning in this study is the establishment of AOPP-mediated senescent cell and animal models. Previous studies usually established senescent endothelial cell model through hydrogen peroxide (H_2_O_2_) or Ang II treatment *in vitro* (31, 43, 44), while *in vivo* vascular senescence model was more commonly established by senescence accelerated mouse/prone (SAMP) model or transgenic mouse model, such as POLG-KO mouse (45–47). We are the first to prove that AOPP stimulation could establish a stable senescent endothelial cell model *in vitro*. In addition, this study demonstrated that AOPPs could also be injected intraperitoneally or intravenously to induce the vascular senescence *in vivo*. AOPPs are the oxidative metabolites found in the human body, so AOPP-induced senescence model is very close to natural conditions (48, 49). Since AOPPs are advanced products with specific receptor such as RAGE, they might exert more lasting and more stable effect than the model established through hydrogen peroxide (H_2_O_2_). AOPPs could also induce cellular senescence in vivo by intraperitoneal or intravenous injection to maintain the consistency of cellular and animal models, while H_2_O_2_ is hard and seldom used in animal model. The commonly used *in vivo* vascular senescence models nowadays were rapid aging mice (SAMP) or transgenic mice model. Therefore, AOPPs stimulation is expected to provide a reliable model for the study of stress-induced premature senescence of endothelial cells, or even other type of vascular cells. This model has not been reported yet, and it is feasible and innovative.

By applying above-mentioned AOPP-treated animal model, we verify the effects of AOPPs on endothelial senescence and the development of vascular disease in ApoE^−/−^ mice fed with high-fat diet in this study. The results proved that AOPPs could effectively induce endothelial senescence *in vivo*, which is one of the important reasons for vascular senescence. AOPPs also increased the aortic plaque area in ApoE^−/−^ mice, which indicated that AOPPs could promote the development of atherosclerosis. ApoE^−/−^ mice injected with AOPPs plus rapamycin showed a decline in the expression of senescence associated protein p16 and a narrow in the aortic plaque area, which implied autophagy activation could attenuate AOPP-induced vascular senescence and might slow down the development of atherosclerosis. Besides, the rapamycin-mediated attenuation of p53 SUMOylation in aortic tissue support the notion that AOPPs induce endothelial and vascular senescence through p53 SUMOylation.

In conclusion, this study provides sufficient evidences *in vitro* and *in vivo* to unveiled the role of AOPPs in inducing endothelial senescence and apoptosis evasion. Our study also indicates that, by binding to RAGE, AOPPs could accelerate endothelial senescence through inhibiting autophagy, and subsequently inducing p53 SUMOylation. These findings help to clarify the role of p53 SUMOylation in the pathogenesis of cellular senescence-mediated vascular diseases, such as atherosclerosis. Furthermore, the finding of p53 SUMOylation at K386 provides a new therapeutic target for diseases associated with endothelial senescence.

## Data Availability Statement

The original contributions presented in the study are included in the article/Supplementary Material, further inquiries can be directed to the corresponding author/s.

## Ethics Statement

The animal study was reviewed and approved by the Animal Care and Use Committee of Southern Medical University.

## Author Contributions

YC, XG, and QH conceptualized and designed the study and interpreted the data. YC, ZL, HC, XinH, XiaH, YL, and QL conducted cellular and animal experiments and generated figures. YC, JW, and QZ conducted IHC and imaging analyses. YC performed statistical analysis. YC and QH wrote the original draft of the manuscript. All authors contributed to review and editing of the manuscript.

## Funding

This work was supported by the National Natural Science Foundation of China Grants (81870210), Guangdong Basic and Applied Basic Research Foundation (2019A1515012022), and President Foundation of Nanfang Hospital, Southern Medical University (2017C020).

## Conflict of Interest

The authors declare that the research was conducted in the absence of any commercial or financial relationships that could be construed as a potential conflict of interest.

## Publisher's Note

All claims expressed in this article are solely those of the authors and do not necessarily represent those of their affiliated organizations, or those of the publisher, the editors and the reviewers. Any product that may be evaluated in this article, or claim that may be made by its manufacturer, is not guaranteed or endorsed by the publisher.

## Supplementary Material

Supplementary Figure 1 The colocalizations of RAGE, LC3, SUMO1, p16 and p21 with endothelial cell marker CD31 by immunofluorescence. Aortas tissue isolated from ApoE^−/−^ mice fed with high-fat diet were paraffin-embedded and the sections were then immunofluorescently stained and observed under confocal microscopy. **(A)** Aortic section were stained with RAGE (red) and CD31 (green). **(B)** Aortic section were stained with LC3 (red) and CD31 (green). **(C)** Aortic section were stained with SUMO1 (red) and CD31 (green). **(D)** Aortic section were stained with p16 (red) and CD31 (green). **(E)** Aortic section were stained with p21 (red) and CD31 (green). Cell nucleus was indicated with DAPI (blue). White arrows indicated the aortic endothelium. Scale bar = 10 μm.
