# Supplementary material for: p53 SUMOylation Mediates AOPP-Induced Endothelial Senescence and Apoptosis Evasion
Source: Front Cardiovasc Med. 2022 Feb 3;8:795747. doi: 10.3389/fcvm.2021.795747 (PMC8850781; doi:10.3389/fcvm.2021.795747)

SUPPLEMENTAL MATERIAL

Unedited Immunoblots

Full unedited gels for Figure 1A

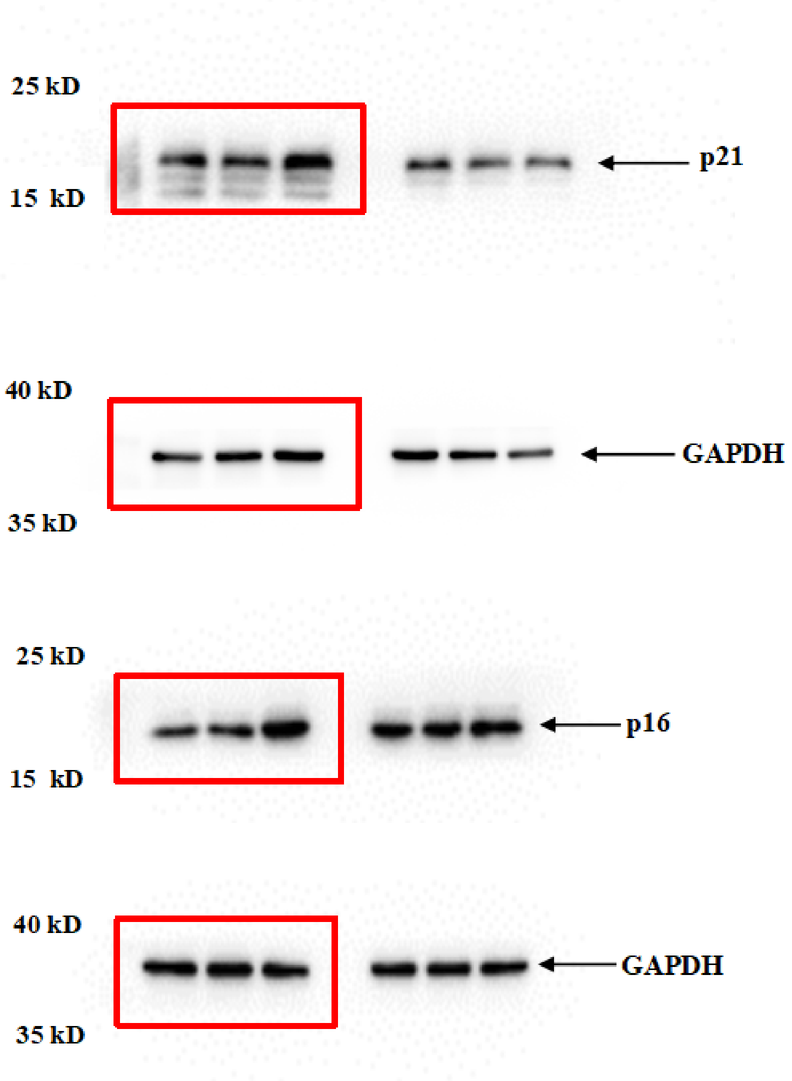

Full unedited gels for Figure 1F

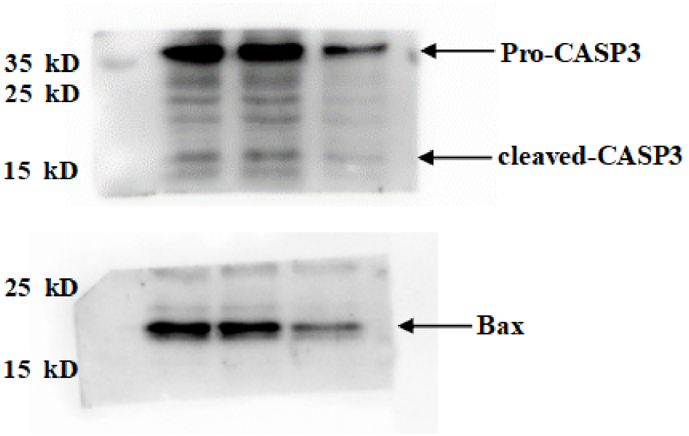

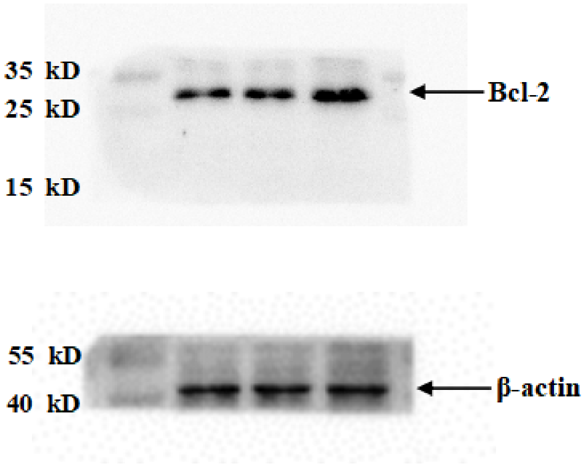

Full unedited gels for Figure 2A

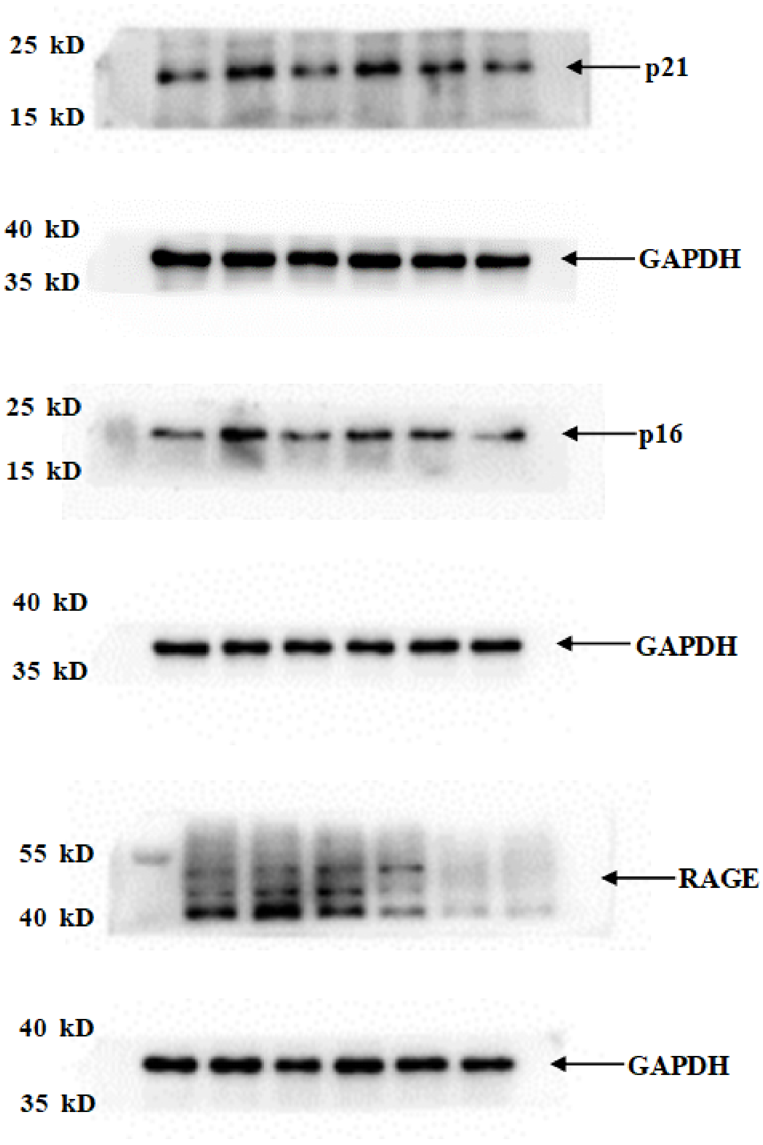

Full unedited gels for Figure 4A

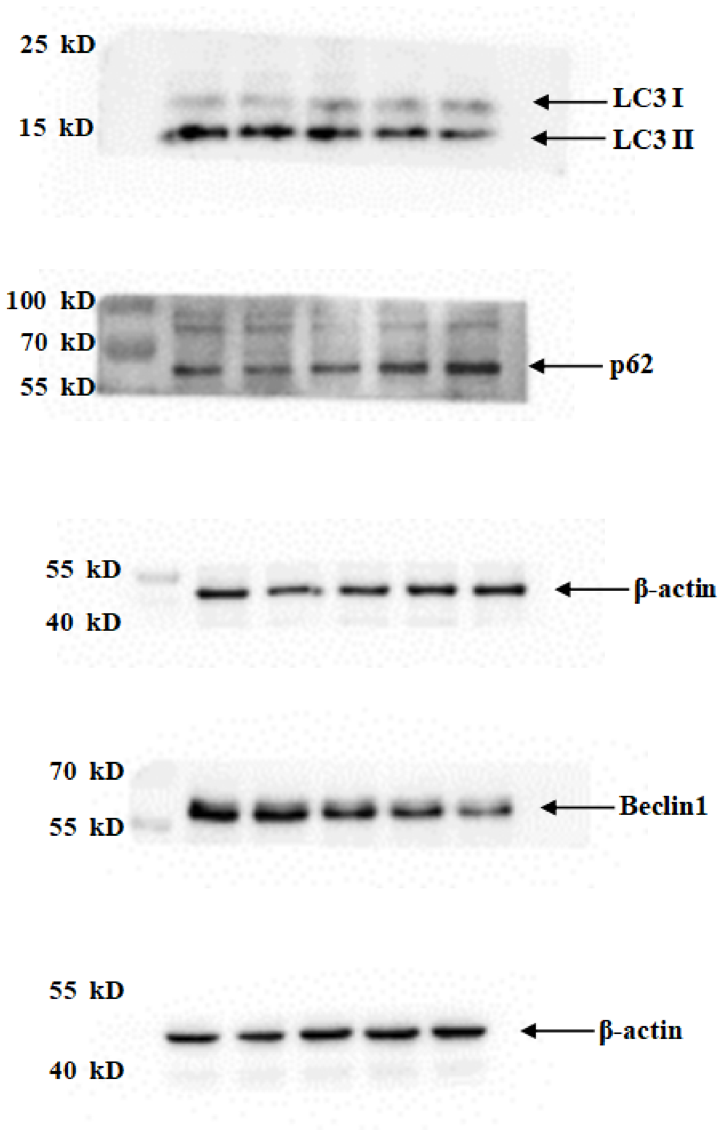

Full unedited gels for Figure 4F

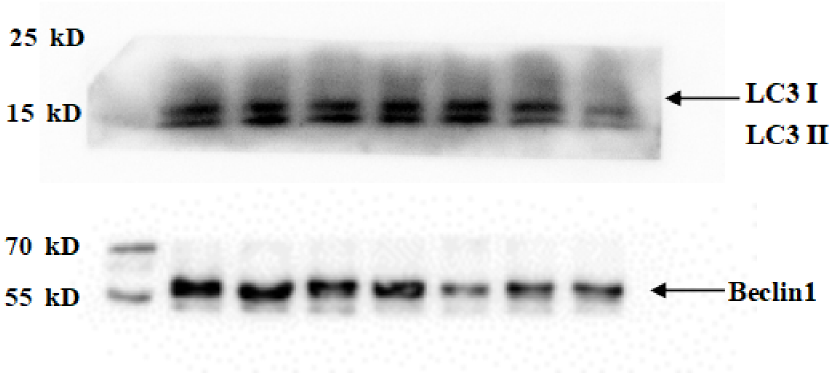

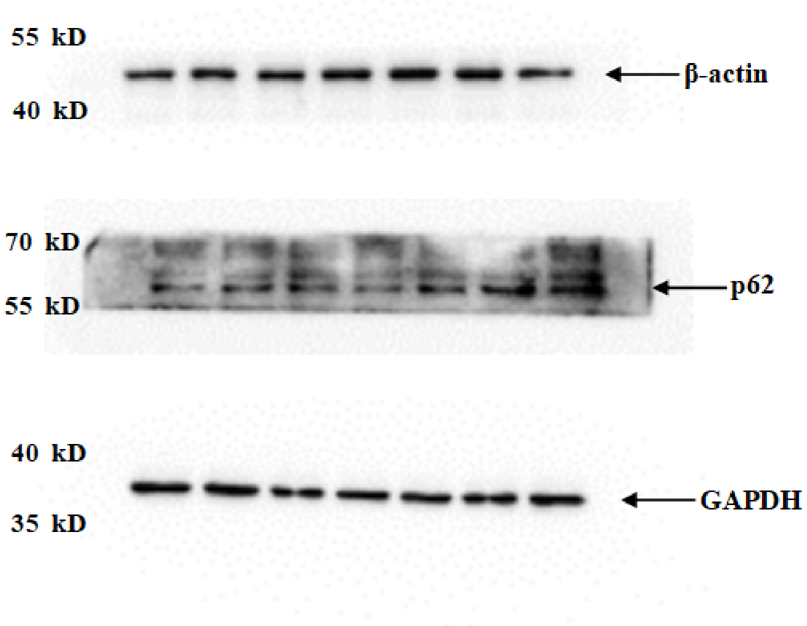

Full unedited gels for Figure 5C

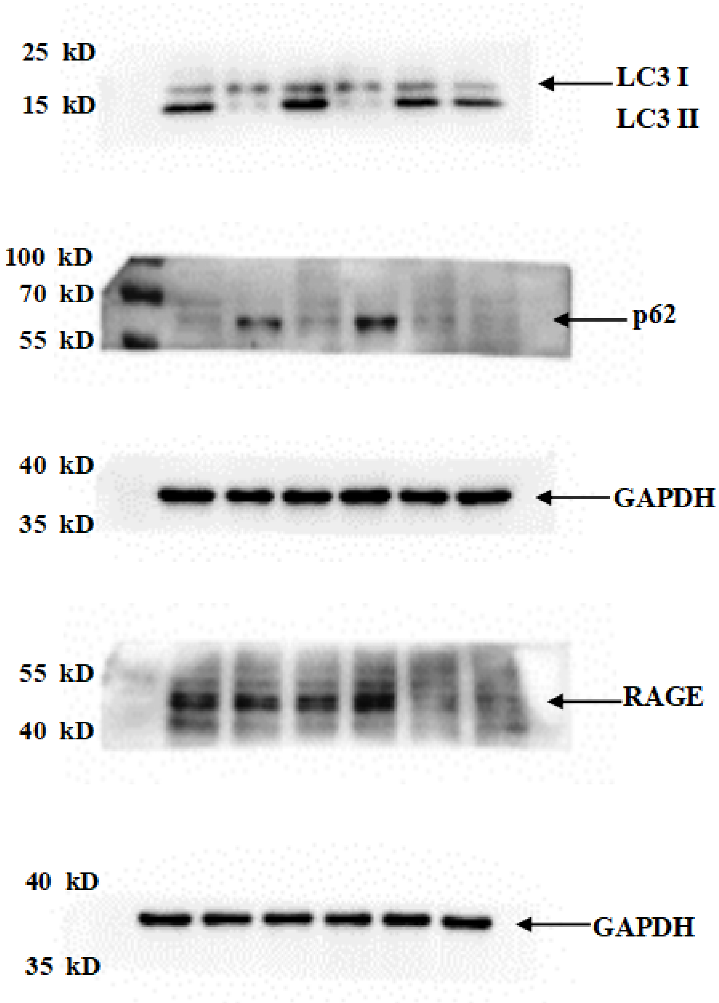

Full unedited gels for Figure 5F

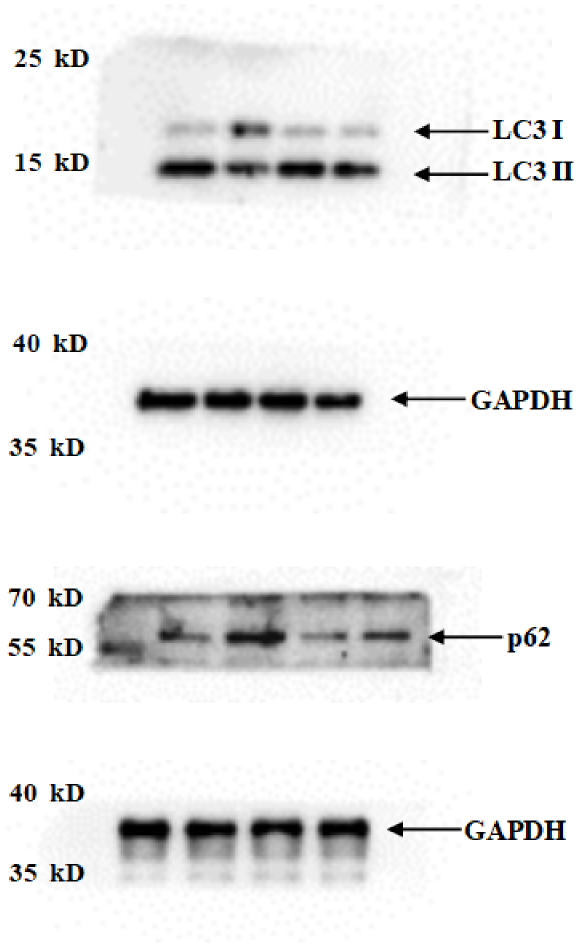

Full unedited gels for Figure 6A

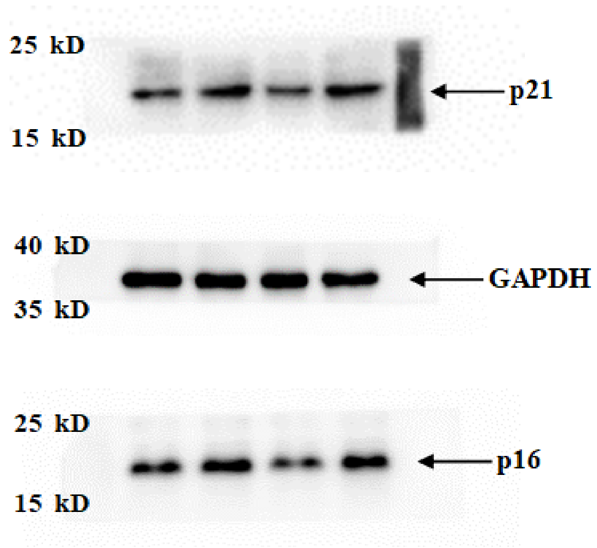

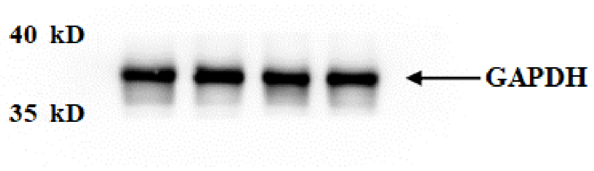

Full unedited gels for Figure 7A

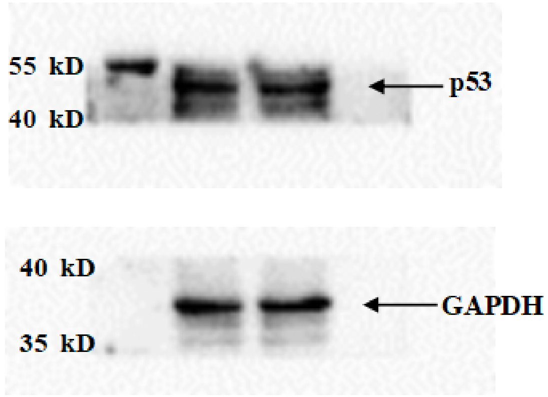

Full unedited gels for Figure 7C

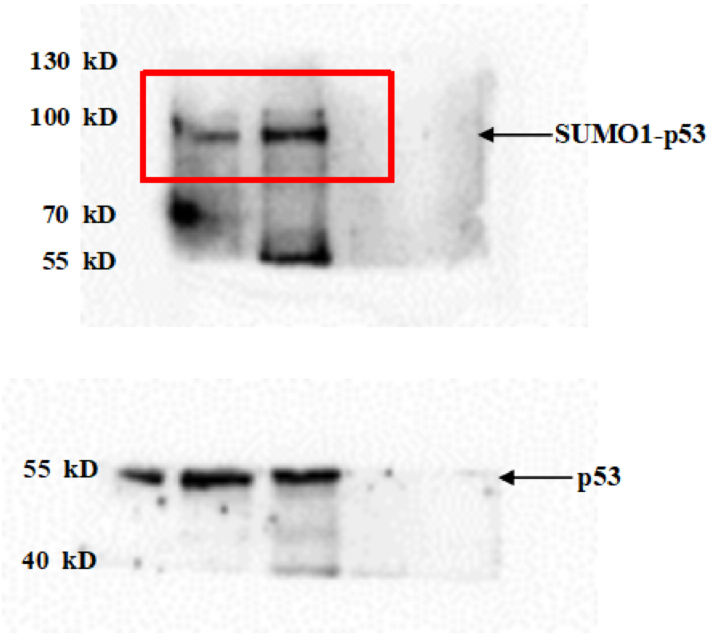

Full unedited gels for Figure 7E

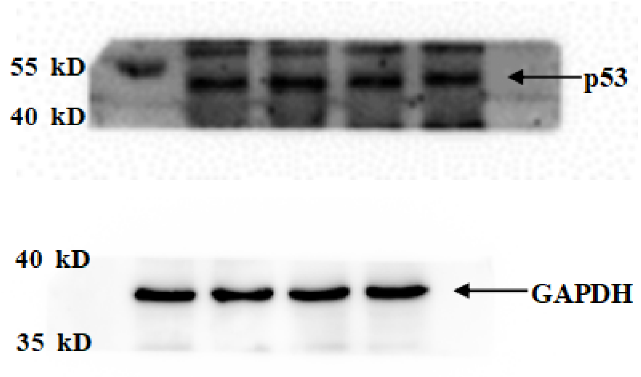

Full unedited gels for Figure 7G

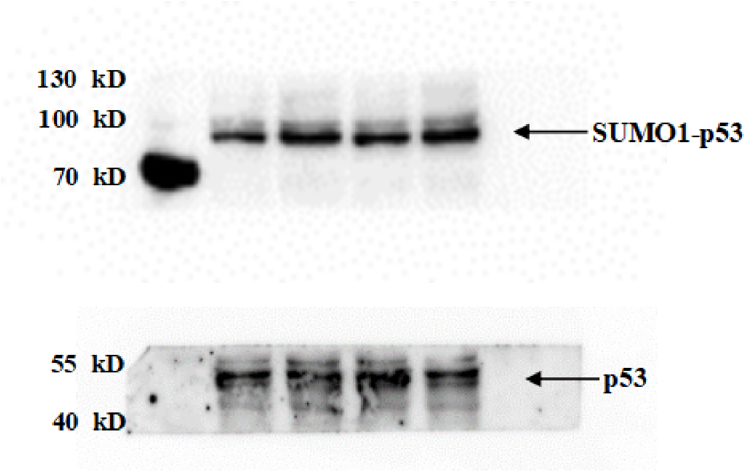

Full unedited gels for Figure 8A

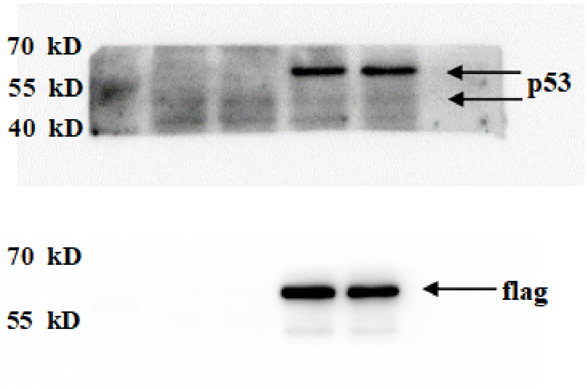

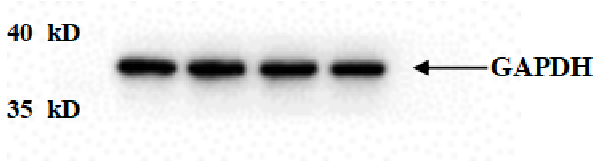

Full unedited gels for Figure 8D

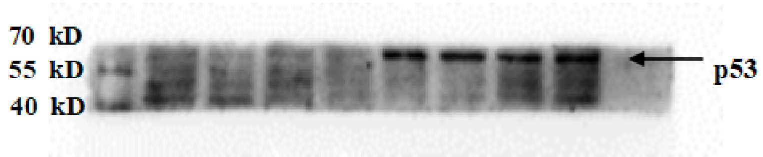

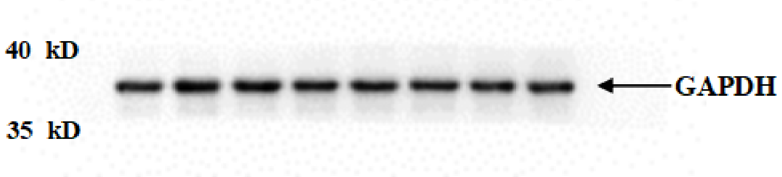

Full unedited gels for Figure 8F

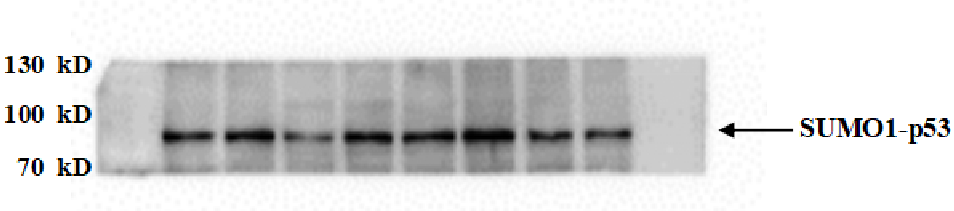

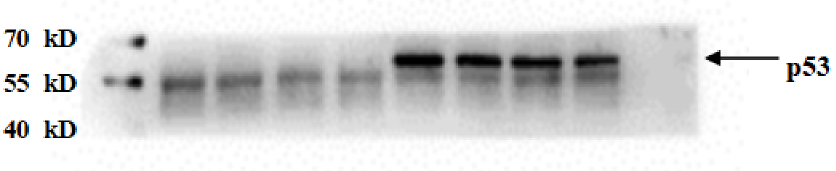

Full unedited gels for Figure 8H

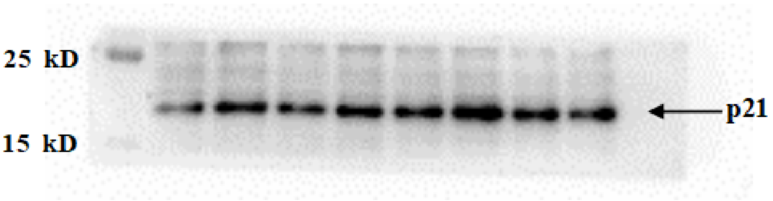

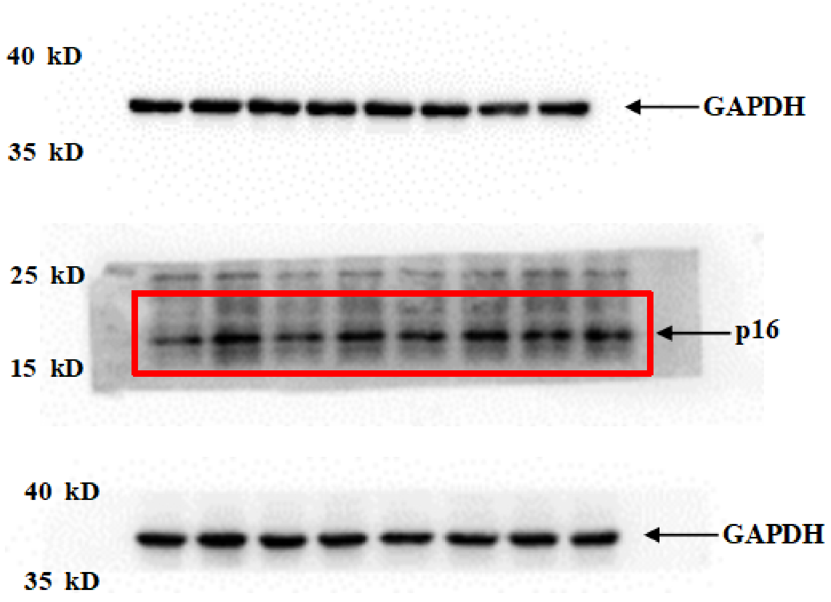

Full unedited gels for Figure 8M

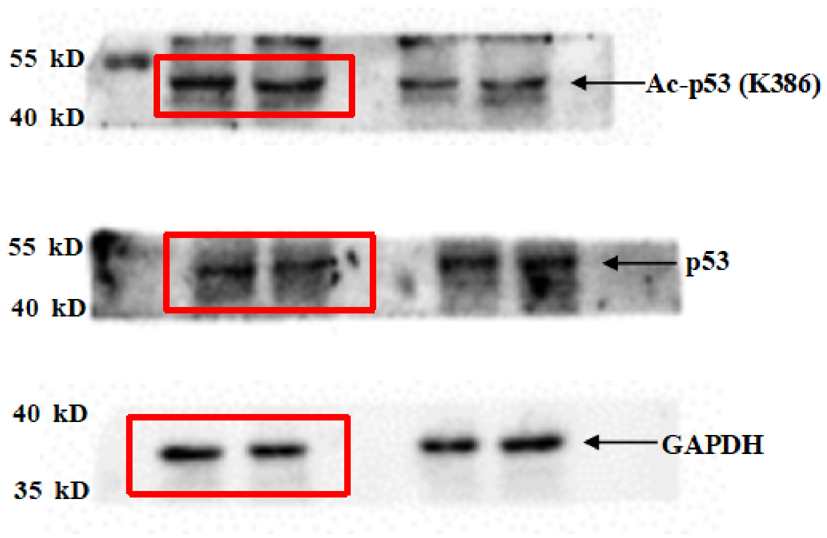

Full unedited gels for Figure 9A

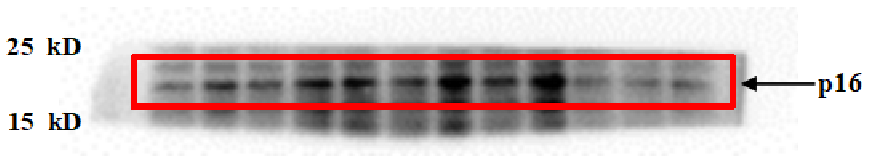

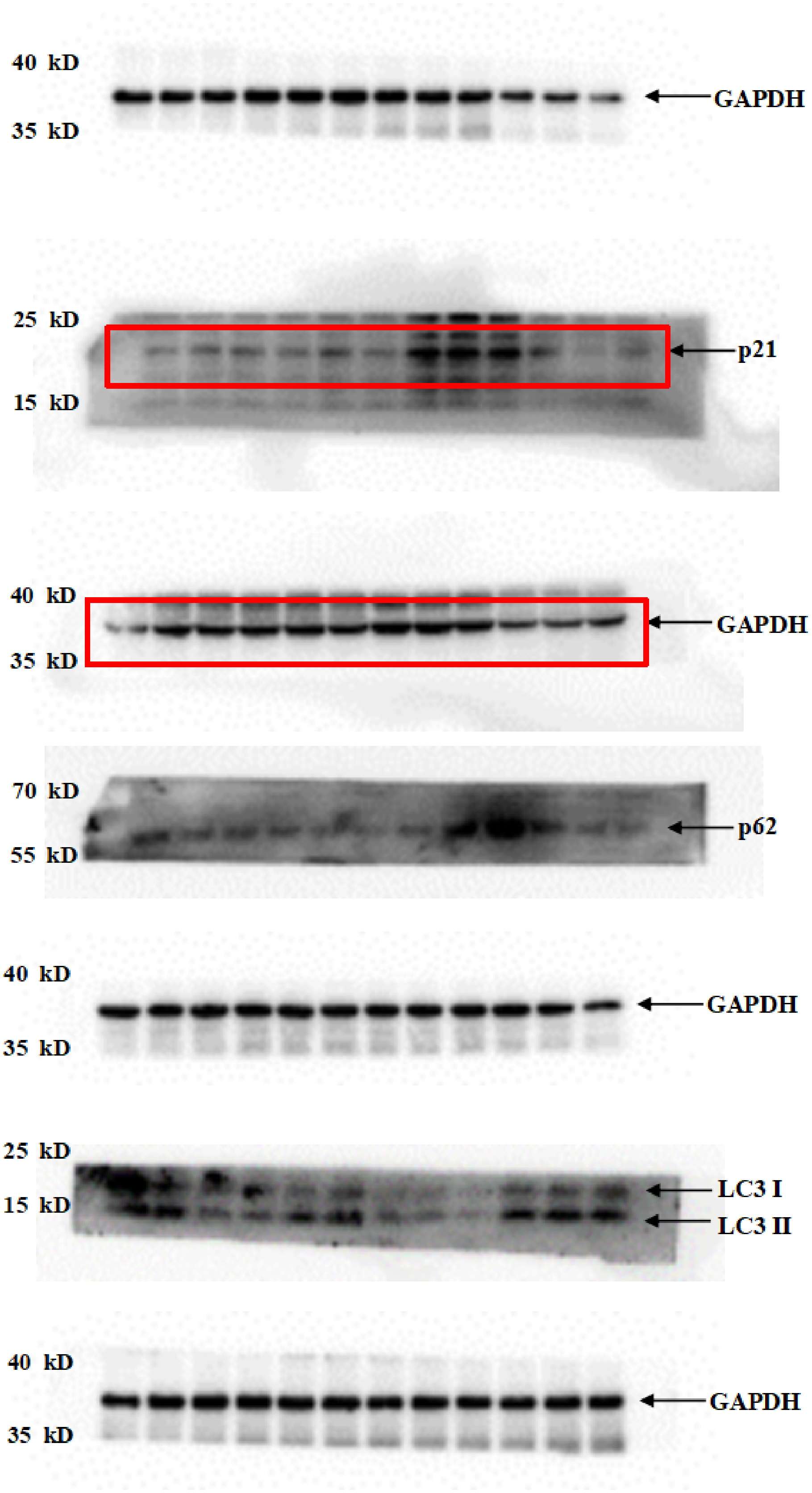
40 kD
35 kD
GAPDH
25 kD
15 kD
p21
40 kD
35 kD
GAPDH
70 kD
55 kD
p62
40 kD
35 kD
GAPDH
25 kD
15 kD
LC3 I
LC3 II
40 kD
35 kD
GAPDH

Full unedited gels for Figure 9F

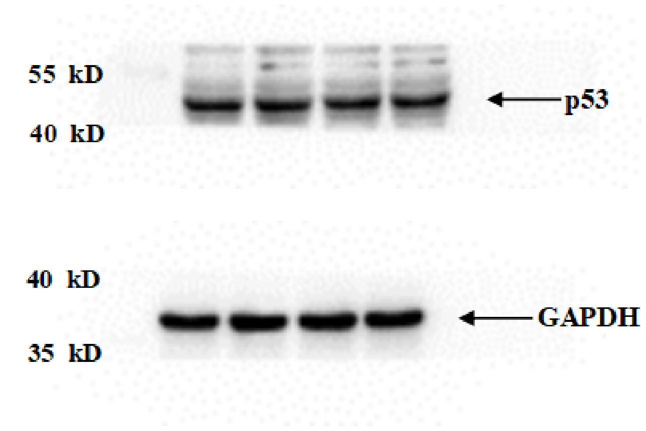

Full unedited gels for Figure 9G

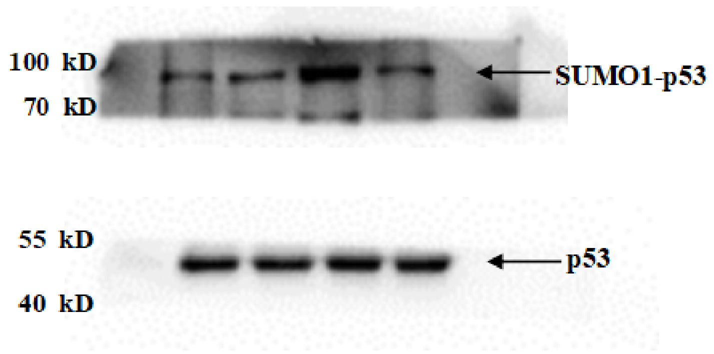

Supplement: Supplementary file 1 [file Data_Sheet_1.pdf]
