# Supplementary material for: p53 SUMOylation Mediates AOPP-Induced Endothelial Senescence and Apoptosis Evasion
Source: Front Cardiovasc Med. 2022 Feb 3;8:795747. doi: 10.3389/fcvm.2021.795747 (PMC8850781; doi:10.3389/fcvm.2021.795747)
Supplement: Supplementary file 2 [file Data_Sheet_2.pdf]

Major Resources Table

Animals (in vivo studies)

Species	Vendor or Source	Background Strain	Sex	Persistent ID / URL

Genetically Modified Animals

	Species	Vendor or Source	Background Strain	Other Information	Persistent ID / URL
**Parent - Male**	Mouse	GemPharmatech Co., Ltd	B6/JGpt-Apoe^{em1Cd82} /Gpt	Apoe KO	http://order.gempharmatech.com/strain/index?keyword=Apoe

Antibodies

Target antigen	Vendor or Source	Catalog #	Working concentration	Persistent ID / URL
RAGE	Abcam	Ab216329	1:1000 for WB<br>1:100 for IHC	https://www.abcam.cn/rage-antibody-epr21171-ab216329.html
p16	ABclonal	A0262	1:1000 for WB<br>1:200 for IHC	https://abclonal.com.cn/catalog/A0262
p21	ABclonal	A2691	1:1000 for WB	https://abclonal.com.cn/catalog/A2691
p53	Immunoway	YT3528	1:1000 for WB	http://www.immunoway.com/Home/22/YT3528
p53	Proteintech	60283-2-Ig	1:50 for IP	https://www.ptgcn.com/products/P53-Antibody-60283-2-Ig.htm
Acetyl-p53 (K386)	Immunoway	YK0017	1:1000 for WB	http://www.immunoway.com/Home/22/YK0017
SUMO1	Immunoway	YT4470	1:1000 for WB<br>1:200 for IHC	http://www.immunoway.com/Home/22/YT4470
LC3A/B	Cell Signaling Technology	12741	1:1000 for WB<br>1:100 for IF<br>1:100 for IHC	https://www.cellsignal.cn/products/primary-antibodies/lc3a-b-d3u4c-xp-rabbit-mab/12741?site-search-type=Products&N=4294956287&Ntt=12741&fromPage=plp&_requestid=914932
p62	ABclonal	A7758	1:1000 for WB	https://abclonal.com.cn/catalog/A7758
Beclin1	Proteintech	11306-1-AP	1:1000 for WB	https://www.ptgcn.com/products/BECN1-Antibody-11306-1-AP.htm
Bcl-2	Immunoway	YT0470	1:1000 for WB	http://www.immunoway.com/Home/22/YT0470
Bax	Immunoway	YT0455	1:1000 for WB	http://www.immunoway.com/Home/22/YT0455
Caspase-3	Cell Signaling Technology	9662	1:1000 for WB	https://www.cellsignal.cn/products/primary-antibodies/caspase-3-antibody/9662?site-search-type=Products&N=4294956287&Ntt=9662&fromPage=plp
RAGE neutralizing antibody	R&D	MAB11451	10 μg/mL for blockade of receptor-ligand interaction	https://www.rndsystems.com/cn/products/human-rage-antibody-176902_mab11451
Mouse IgG	ABclonal	AC011	1:50 for IP	https://abclonal.com.cn/catalog/AC011
GAPDH	Beijing Ray	RM2002	1:5000 for WB	http://www.rayantibody.com/index.php?c=product&a=type&tid=13

β-actin	Beijing Ray	RM2001	1:5000 for WB	http://www.rayantibody.com/index.php?c=product&a=type&tid=13
Secondary antibodies	Beijing Ray	RM3001<br>RM3002	1:5000 for WB	http://www.rayantibody.com/index.php?c=product&a=type&tid=15
DAPI	Solarbio	C0065	for IF	http://m.solarbio.com/goods-258.html
Alexa Fluor 594	Immunoway	RS3611	1:200 for IF	http://www.immunoway.com/Home/22/RS3611

DNA/cDNA Clones

Clone Name	Sequence	Source / Repository	Persistent ID / URL
TP53(NM_000546(K386R)) cDNA	gcagatcgaattaagcttgggctgcaggtcgactctagaggatcccgccaccat<br>ggaggagccgcagtcagatcctagcgtcgagcccctctgagtcaggaaacatt<br>ttcagacctatggaaactacttcctgaaaacaacgttctgtcccccttgccgtccc<br>aagcaatggatgatttgatgctgtccccggacgatattgaacaatggttcactga<br>agacccaggtccagatgaagctcccagaatgccagaggctgctccccccgtggc<br>ccctgcaccagcagctcctacaccggcggcccctgcaccagccccctcctggcc<br>cctgtcatcttctgtcccttcccagaaaacctaccagggcagctacggtttccgtct<br>gggcttcttgcattctgggacagccaagtctgtgacttgcacgtactcccctgccc<br>tcaacaagatgttttgccaactggccaagacctgccctgtgcagctgtgggttga<br>ttccacacccccgcccggcacccgcgtccgcgccatggccatctacaagcagtc<br>acagcacatgacggaggttgtgaggcgctgcccccaccatgagcgctgctcaga<br>tagcgatggtctggcccctcctcagcatcttatccgagtggaaggaaatttgcgtg<br>tggagtatttggatgacagaaacacttttcgacatagtgtggtggtgccctatgag<br>ccgcctgaggttggctctgactgtaccaccatccactacaactacatgtgtaaca<br>gttcctgcatgggcggcatgaaccggaggcccatcctcaccatcatcacactgga<br>agactccagtggtaatctactgggacggaacagctttgaggtgcgtgtttgtgcct<br>gtcctgggagagaccggcgcacagaggaagagaatctccgcaagaaagggga<br>gcctcaccacgagctgcccccagggagcactaagcgagcactgcccaacaaca<br>ccagctcctctccccagccaaagaagaaaccactggatggagaatatttcaccc<br>ttcagatccgtgggcgtgagcgcttcgagatgttccgagagctgaatgaggcctt<br>ggaactcaaggatgcccaggctgggaaggagccagggggggagcagggctcac<br>tccagccacctgaagtccaaaaagggtcagtctacctcccgccataaaaaactc<br>atgttcaggacagaagggcctgactcagacggtatggactacaaggatgacgat<br>gacaaggattacaaagacgacgatgataaggactataaggatgatgacgacaa<br>agctagctaactgtggaatgtgtgtcagttagggtgtggaaagtccccaggctcc<br>ccagcaggcagaagtatgcaaagcatgcatctcaattagtcagcaaccaggtgt<br>ggaaagtccccaggctccccagcaggcagaagtatgcaaagcatgcatctcaat<br>tagtcagc	Genechem	Does not apply
TP53 Fwd Primer	aggtcgactctagaggatcccgccaccatggaggagccgcagtcagatc	Genechem	Does not apply
TP53 Rev Primer	tccttgtagtccataccgtctgagtcaggcccttctgtcctgaac	Genechem	Does not apply
GV314 (Adenoviral Vector)	https://www.genechem.com.cn/index/supports/zaiti_info.html?keywords=GV314	Genechem	https://www.genechem.com.cn/index/supports/zaiti_info.html?keywords=GV314

Cultured Cells

Name	Vendor or Source	Sex (F, M, or unknown)	Persistent ID / URL
Human umbilical vein endothelial cells	ScienCell	unknown	https://www.sciencellonline.com/human-umbilical-vein-endothelial-cells.html

Data & Code Availability

Description	Source / Repository	Persistent ID / URL

Other

Description	Source / Repository	Persistent ID / URL
Endothelial cell medium	ScienCell	https://www.sciencellonline.com/endothelial-cell-medium.html
Endothelial cell growth supplement	ScienCell	https://www.sciencellonline.com/endothelial-cell-growth-supplement.html
Fetal bovine serum	ScienCell	https://www.sciencellonline.com/fetal-bovine-serum.html
HBSS	Gibco	https://www.thermofisher.cn/order/catalog/product/14025092?SID=srch-srp-14025092#/14025092?SID=srch-srp-14025092
Cell Counting Kit-8	Invigentech	http://www.ivigt.com/content/?212.html
Matrigel Matrix	Corning	https://ecatalog.corning.com/life-sciences/b2c/US/en/Surfaces/Extracellular-Matrices-ECMs/Corning%C2%AE-Matrigel%C2%AE-Matrix/p/corningMatrigelMatrix
Collagen Ⅰ, Rat tail	Corning	https://ecatalog.corning.com/life-sciences/b2c/US/en/Surfaces/Extracellular-Matrices-ECMs/Corning%C2%AE-Collagen/p/corningCollagen
FITC-dextran	Sigma-Aldrich	https://www.sigmaaldrich.cn/CN/zh/product/sigma/fd40s?context=product
Rapamycin	MedChemExpress	https://www.medchemexpress.cn/Rapamycin.html
3-MA	Selleck	https://www.selleck.cn/products/3-methyladenine.html
Protein A+G Agarose	Beyotime	https://www.beyotime.com/product/P2012.htm
Senescence β-galactosidase staining kit	Beyotime	https://www.beyotime.com/product/C0602.htm
Annexin V-FITC/PI apoptosis detection kit	BestBio	http://www.bestbio.com.cn/goods.php?id=53
siRNA-Mate	GenePharma	https://www.genepharma.com/show.php?ctype=0&coupid=568&cateid=111
