# Supplementary figures and images for: p53 SUMOylation Mediates AOPP-Induced Endothelial Senescence and Apoptosis Evasion
Source: Front Cardiovasc Med. 2022 Feb 3;8:795747. doi: 10.3389/fcvm.2021.795747 (PMC8850781; doi:10.3389/fcvm.2021.795747)

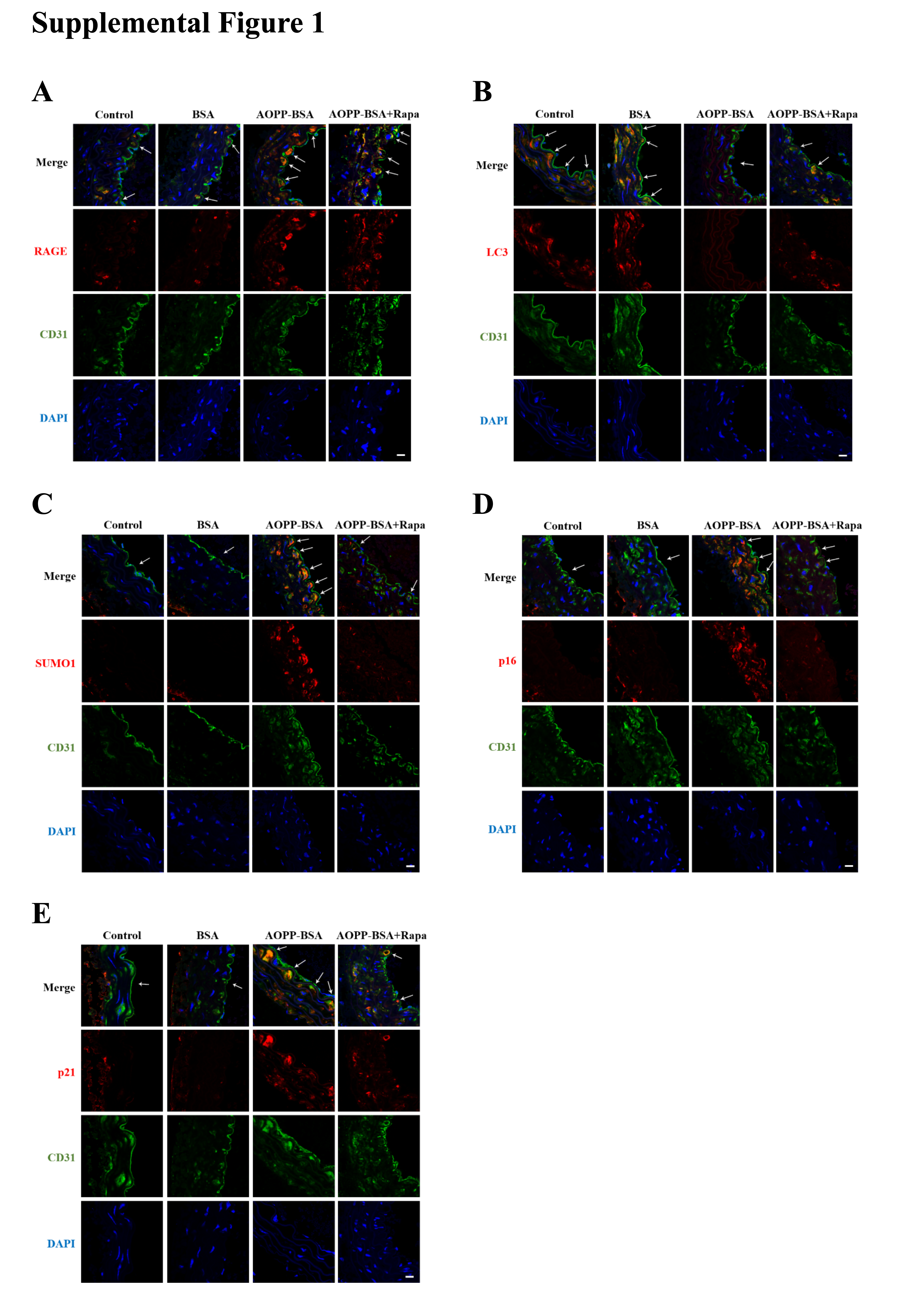

Supplement: Supplementary Figure 1 — The colocalizations of RAGE, LC3, SUMO1, p16 and p21 with endothelial cell marker CD31 by immunofluorescence. Aortas tissue isolated from ApoE−/− mice fed with high-fat diet were paraffin-embedded and the sections were then immunofluorescently stained and observed under confocal microscopy. (A) Aortic section were stained with RAGE (red) and CD31 (green). (B) Aortic section were stained with LC3 (red) and CD31 (green). (C) Aortic section were stained with SUMO1 (red) and CD31 (green). (D) Aortic section were stained with p16 (red) and CD31 (green). (E) Aortic section were stained with p21 (red) and CD31 (green). Cell nucleus was indicated with DAPI (blue). White arrows indicated the aortic endothelium. Scale bar = 10 μm. [file Image_1.TIFF]
